# Long‐term labour market and economic consequences of school exclusions in England: Evidence from two counterfactual approaches

**DOI:** 10.1111/bjep.12487

**Published:** 2022-02-09

**Authors:** Joan E. Madia, Ingrid Obsuth, Ian Thompson, Harry Daniels, Aja L. Murray

**Affiliations:** ^1^ Department of Sociology Nuffield College University of Oxford UK; ^2^ FBK‐IRVAPP Trento Italy; ^3^ Clinical Psychology Department University of Edinburgh UK; ^4^ Department of Education University of Oxford UK; ^5^ Department of Psychology University of Edinburgh UK

**Keywords:** inverse probability treatment weighting, NEET, propensity score analysis, school exclusion, unemployment

## Abstract

**Background:**

Previous research suggests that school exclusion during childhood is a precursor to social exclusion in adulthood. Past literature on the consequences of school exclusion is, however, scarce and mainly focused on short‐term outcomes such as educational attainment, delinquency, and mental health in early adolescence. Moreover, this evidence is based primarily on descriptive and correlational analysis, whereas robust causal evidence is required to best inform policy.

**Aims:**

We aimed to estimate the mid‐to‐long‐term impact of school exclusion on labour market and economic outcomes.

**Sample:**

The sample included 6,632 young people who at the age of 25/26 in the year 2015 participated in the Next Steps survey of whom 86 were expelled from school and 711 were suspended between the ages of 13/14 and 16/17.

**Method:**

Using high quality existing longitudinal data, we utilized four approaches to evaluate the impact of school exclusion: logistic regression‐adjustment models, propensity score matching, school fixed‐effects analysis, and inverse propensity weighting. The latter two counterfactual approaches were used to estimate causal effects.

**Results:**

We found that school exclusion increased the risk of becoming NEET at the age of 19/20, and then remaining economically inactive at the age of 25/26, as well as experiencing higher unemployment risk and earning lower wages also at the age of 25/26.

**Conclusion:**

School exclusion has pervasive negative effects into adulthood. Policy interventions should focus on both prevention and mitigating its negative effects. Interventions aimed at re‐integrating excluded individuals into education or vocational training could be key in reducing the risk of poor socio‐economic outcomes and social exclusion.

## Introduction and Background

School exclusion is used as a disciplinary instrument by schools to respond to ‘disruptive’ or ‘challenging’ pupil behaviour (Daniels & Cole, 2010), which may include anything from persistent disruptive behaviour to physical assault and sexual misconduct (Timpson, 2019). In England, the headteacher of a school can *temporarily exclude* students from school for a fixed period of time, typically from 1 to 5 days with a legal maximum of 45 days a year, or they can *permanently exclude* students from the school. Permanent exclusion can leave students without education until a new school accepts them or, as noted by the Children’s Commissioner for England (2019), may push parents to decide to home‐educate their children. Recently, the UK Department for Education (DfE, 2021a) suggested alternative terminology in lieu of temporary and permanent exclusion, namely these were to be replaced by ‘suspension’ and ‘expulsion’ from school, respectively. Subsequently, the UK Department for Education (DfE, 2021b) updated the terminology to ‘suspension’ and ‘permanent exclusion’, respectively. Herewith we will utilize the latter terminology and refer to them as school exclusion when referring to both.

In recent years, school exclusion has steadily increased in England in contrast to the rest of the United Kingdom. In 2011, for example, the rate of permanent exclusion at primary and lower secondary education was 0.06% while in 2018 it increased to 0.12%, representing a doubling in only 7 years (Timpson, 2019). This, indeed, has raised major concerns among politicians and policy makers and gained considerable attention in the public debate (EPI, 2019; Graham, White, Edwards, Potter, & Street, 2019; Timpson, 2019), particularly because permanent exclusions from school may not be effective in correcting poor behaviour. Rather, they are liable to worsen the situation of vulnerable students who are already at risk of poor educational and occupational attainment, or of social exclusion more broadly (Parsons, 2018). For example, systemic pressures have led to a disproportionate number of school exclusions of students with social emotional and mental health special educational needs (Thompson, Tawell, & Daniels, 2021). Students eligible for free school meals and from Black Caribbean and Gypsy/Roma ethnic backgrounds also have much higher exclusion rates (DfE, 2021). Indeed, Graham et al. (2019) point out that many of the students most vulnerable to exclusion face multiple layers of disadvantage.

Understanding the consequences of school exclusion is important for designing policies informed by knowledge of its individual as well as societal costs and to mitigate its adverse impacts. However, empirical evidence on the impact of school exclusion remains rather scarce and largely limited to descriptive and correlational analyses. Further, the majority of the literature on consequences of school exclusion has been developed in the United States, and research in other countries, including the United Kingdom is more lacking. Evidence from the United States suggests a direct association between school exclusion and school retention, dropout, anti‐social behaviour, and delinquency (*see for example,* Costenbader & Markson, 1998; Skiba & Knesting, 2001; Perry & Morris, 2014). Also, a more recent contribution by Rosenbaum (2020) indicates that there could be a causal relationship between exclusions and these outcomes. For example, she used matching methods to show that pupils who experienced school suspensions were more likely to be involved with the justice system and have lower educational attainment. However, the United States has specific institutional features in the education and justice system, as well as exclusion criteria varying across each State, which may limit the generalizability of findings to other countries.

The small number of studies that have addressed this issue in the United Kingdom shows some potentially important associations as well. In the short term, that is, one year following the exclusion, excluded students were reported to have poor academic outcomes (DfE, 2011). They were also reported to be at an increased risk for substance use and delinquency (Timpson, 2019) as well as engaging in self‐harm (McAra & McVie, 2010; McCrystal, Percy, & Higgins, 2007). In the medium to longer term, young people who were excluded were reported to be less likely to be employed in early adulthood (DfE, 2011). For example, in their retrospective study, Spielhofer et al. (2009) found that truancy and school exclusion were two important predictors of becoming NEET. Similarly, findings from Massey (2011), who followed a group of young people who had experienced exclusion, suggested that within two‐three years after their exclusion, half of excluded students transitioned to a NEET status, compared to the UK average of around 13%.

Taken together, children and adolescents who are subject to suspension or permanent exclusion from school seem to be at considerably greater risk of behavioural, health‐related, educational, and employment difficulties. However, the majority of these results are based on correlational and descriptive analyses. One exception for England is an unpublished study conducted by Sutherland and Eisner (2014) using the Next‐steps (LYPSE) data and quasi‐experimental matching methods. They focused on the risk of becoming NEET at the age of 19 following suspension and found that those students who were suspended at the age of 15/16 were between 0.058 and 0.126 percentage points more likely to become NEET at age 19. Their findings supported what has been evidenced in past correlational research. However, the longer term causal impact of exclusion beyond adolescence remains to be examined. The difficulties faced by excluded students might be even larger at later stages of their life, with the accumulation of disadvantage. It is well known in the economic literature, youth unemployment and inactivity are linked to higher risk of unemployment, earning loses, and higher dependency on the welfare system in adulthood (Gregg, 2001; Gregg & Tominey, 2005).

Building on previous research, we thus use high quality longitudinal data and several complementary approaches to assess whether permanent exclusion from school appears to act as a causal factor in poorer socio‐economic outcomes for individuals in the medium and long term. We focused on the likelihood of becoming NEET (i.e., not in education, employment, or training), unemployment and being in low paid jobs as a consequence of permanent exclusion 3–4 (age 19/20) and 8–9 years (age 24/25) following the exclusion. Testing this is challenging because different sources of endogeneity can bias results. That is, a range of individual and social background characteristics are likely to affect both the risk of school exclusion and socioeconomic outcomes in early adulthood. Therefore, to identify the casual effects of school exclusion, it is necessary to find a counterfactual group of students who have similar background characteristics of those excluded but have not experienced this event. To achieve this, we adopted several complementary approaches: regression adjustment, school fixed effects, propensity score matching, and inverse probability treatment weighting. Based on previous evidence available thus far, we hypothesized that those who have experienced school exclusion will be at an increased risk of becoming NEET, economically inactive, unemployed, or in low paid jobs.

## Methods

### Data/participants

We used data from the ‘*Next steps*’ survey also known as the ‘*Longitudinal Study of Young People in England*’ (LSYPE), which is a prospective panel data set made up of eight waves collected annually from age 13/14 until age 19/20, with another measurement wave at age 25/26. The sample comprises of individuals born between 1989 and 1990. In the first five waves (ages 13/15 to 15/16), interviews were conducted with students and their parents, collecting information about family background, educational trajectories, attitudes towards education, aspirations, and plans. Subsequently, in waves 6 and 7 (ages 18/19 to 19/20), only the young people were interviewed regarding attitudes towards education and employment, future plans, and their current activities. Five years later, all the young participants of the original survey were re‐contacted in order to collect information on their demographic transitions to adulthood, employment experiences, well‐being, and health (wave 8). Our analytic sample consisted of young people who attended a maintained school^1^ during compulsory education between the years 2004–2008 (at the age of 13/14–16/17; waves 1,2,3, and 4) and were subsequently re‐interviewed at the age of 19/20 (wave 7; year 2010) and 25/26 (wave 8; year 2015). In total, we identified 6632 respondents of whom 711 were temporarily suspended (10.7%, of whom 38% were girls) and 86 expelled from school (1.3%, of whom 40% were girls) once or more times from wave 1 to 4 (between ages 13/14 and 16/17).

### Exposure variable

We focused primarily on the impact of permanent exclusions because the data related to suspensions was incomplete. Specifically, it did not include information about the length of suspensions. Given that in the United Kingdom it is possible to suspend students anywhere from a few hours/one day all the way to four weeks or more, not having this information rendered the definition of the latter category difficult. Including all of the students who would have been suspended (at any length) into the treatment group might have generated a compositional effect. Yet, arguably one relatively short‐term suspension may have a different impact on outcomes than even one relatively long suspension.

We classified students whose parents/guardians reported their exclusions from the age of 13/14 until the age of 16/17 into (a) those who never experienced a suspension or permanent exclusion, (b) those who were suspended, and then (c) those who were expelled from school. This follows the classification adopted by the Department of Education in their reports (*see for example* DfE, 2011). We also created a binary variable representing the treatment status of those who were expelled from school and the control status of those who never experienced any suspension or permanent exclusion. When this variable was employed in the counterfactual models, those suspended from school were removed from the analysis. Our assumption is that suspended students represent a different kind of treatment and thus they cannot be used as control units in the counterfactual analysis. In other words, if we include the suspended students in the reference category, our control group would be characterized by the presence of defiers, that is, units that do not behave in accordance with the hypothetical treatment assignment (Angrist, Imbens, & Rubin, 1996; Balke & Pearl, 1993), making impossible the identification of the potential outcome in the absence of exclusion events in our treatment group.

### Outcome variables

In terms of outcome variables, we focused on self‐reported transitions to labour market, economic activities, and economic well‐being. We examined the *risk of being NEET* at age of 19/20 and then again at the age of 25/26. As regards the NEET definition at age 25/26, we considered someone as such if they reported to be unemployed, not in education or training but also if they were not looking actively for a job (i.e., an economic inactive respondent). We examined whether respondents had *ever been employed*; the probability of *being unemployed* at the time of the interview; and whether respondents were *experiencing economic hardship*. We also examined the employment conditions of those who reported being employed at age 25/26, considering their gross monthly wages, the probability of being in a routine occupation, having a zero‐hours contract, working full‐time, and being in a job where some specific skills are required. In this way, we also assessed the potential disadvantages for those who experienced a school exclusion event but managed to enter the labour market. All these outcome variables were dichotomized except for the monthly gross wage which was log‐transformed and retained as a continuous outcome.

### Covariates

Estimating the consequences of school exclusion is challenging. As previously mentioned, school exclusions are not random events. In general, students with poor behaviour at school are more likely to come from disadvantaged families, live in deprived areas, and attend schools with poorer resources. All these factors increase the probability of misbehaving at school but also having poor educational and employment outcomes during adolescent and early adulthood. Therefore, it is important to make never excluded and excluded pupils as similar as possible. In this regard, LYPSE data provide a comprehensive set of control variables regarding respondents’ demographic characteristics and social background information during childhood/adolescence. We controlled for a set of variables that past research suggested to be strong predictors of permanent exclusion from school (Strand & Fletcher, 2014) and are also associated in the literature with lower labour market prospects (Holmes, Murphy, & Mayhew, 2019). We included sex, the number of siblings, and other adult members in the household (both continuous); an ethnicity dummy variable which identified black Caribbean respondents, that is, an ethnic group more likely to be expelled from schools; whether English was the main language of the household; mother’s age which is typically positively associated with children’s cognitive development; whether the respondent grew up in single parent household; whether parents were in contact with social services due to their child’s behaviour at home or school; and whether respondents were identified as having Special Education Need (SEN) when they were attending mandatory education. These last two variables should capture poor behaviour at school and higher risk of school exclusion. Finally, we included two indices that capture family resources and contextual disadvantages in the neighbourhood area of residence. First, a ‘Socio‐Economic Status index’ (SES) derived from a principal component analysis using highest parental educational attainment, parental class (obtained from the NS‐SEC classification), and housing tenure status. Second, the ‘Income Deprivation Affecting Children index’ (IDACI) which measures the proportion of children under the age of 16 who live in low‐income households in the local area of residence.

### Analytical procedures

We relied on several complementary approaches to account for different sources of potential bias. We first ran logistic regressions for the binary outcomes and OLS regressions for the log of gross monthly wages applying three different specifications: (a) an unconditional regression (i.e., without any control variables) to describe the raw difference in means by school exclusion experiences; (b) conditional regressions included the full set of socio‐demographic and student behaviour covariates described above to account for students’ characteristics and background of origin (c) a school‐fixed effect approach aimed at removing any (constant) unobserved heterogeneity at the school level and tackling self‐selection into poor/rich schools. This latter specification is particularly important to reduce selection bias since vulnerable and disadvantaged students with challenging behaviour are more likely to attend poor schools with a lack of experienced teachers and resources to deal with disruptive situations in classes (Allen & Sims, 2018), increasing the likelihood of excluding these pupils from school as a less costly intervention for preserving the learning environment (Parsons, 2018). Moreover, as our main goal was to provide better evidence for a causal interpretation of the consequences of school exclusion, we also employed Propensity Score Matching (PSM) (Rosenbaum & Rubin, 1983) and Inverse Probability of Treatment Weighting (IPTW) (Robins, Hernán, & Brumback, 2000) as a second approach following a counterfactual logic. PSM has the advantage of balancing the treatment and control groups in a more flexible manner than common regression adjustment methods. It also restricts the estimation to the group of observation within an area of data in which there is a ‘common support’ between observations rather than extrapolating across the sample (Guo & Fraser, 2015). We employed a propensity score one‐to‐one and one‐to‐three nearest neighbour matching estimators with replacement using Leuven and Sianesi's (2003) procedure.^2^ However, as recently noted by Abadie and Imbens (2016), standard propensity score methods usually have larger standard errors due to the fact that the propensity score, used for matching individuals, is itself estimated prior to the treatment‐control groups comparison, thus affecting the large sample distribution of PSM estimator. Therefore, to address this concern, we also employed Abadie and Imbens’ suggested standard errors adjustment.

Moreover, it has been recently argued that PSM, in its attempt to reproduce a randomized experiment, might increase imbalance between control and treatment groups and model dependence due to the impossibility of determining the exact moment when pruning should be stopped (King & Nielsen, 2019). As a solution to this issue, King and Nielsen propose using multivariate distance or exact coarsened matching, which we also employed as robustness check in the Appendix S1. However, matching methods might be less precise when sample sizes are small due to the fact a lot of data can be discarded. Given this potential issue and the fact that our number of treated cases is limited (i.e., our treatment is a rare event in the population), we also employed IPTW as a complementary approach. In this case, the estimated PS are not used to prune the sample but to re‐weight it on the basis of their inverse probability of receiving the treatment, rendering the two groups as similar as possible without any loss of data.^3^ These two counterfactual approaches rely on the assumption that all the potential differences between treatment and control group have been captured by the set of covariates included in the propensity score model and its functional form has been also correctly specified (Cerulli, 2015). For this second part of the analysis, we restricted the sample to individuals who never experienced a suspension/exclusion (i.e., the control group, *D* = 0) and those who experienced a permanent exclusion from school (i.e., the treatment group, *D* = 1). Finally, these counterfactual models were complemented with a series of sensitivity analyses to assess the extent to which results could be negated due to omitted variables. For this robustness check we employed the Mantel–Haenszel bounds for our nonlinear models (Becker & Caliendo, 2007) and the Rosenbaum bounds (DiPrete & Gangl, 2004) for the earnings outcome.

### Missingness and attrition

To deal with non‐random attrition, the data release provides attrition and non‐response weights, which we used in our empirical analysis following the recommendation provided in the technical documents of the Next Step survey and Anders (2012). In addition, we also compared the social background characteristics of our sample before and after selecting those who have remained in the last wave. Although we found that boys who grow up in a disadvantaged background and experienced school exclusion events were more likely to not participate in wave 8, these percentage differences are not substantively different among the two samples.^4^

## Results

Descriptive statistics on social background characteristics by school exclusion events are presented in Table 1. There was substantial variation in the probability of being expelled from school by social background. First, this group of students were more likely to be boys from disadvantaged backgrounds (40% of the total were girls compared to 58% girls in the never excluded group). Their parents had a lower SES, the household was usually single parent, and they were more likely to live in deprived neighbourhoods. In terms of ethnicity, 13% were from a black Caribbean background compared to 5% in the suspended group and 3% in the never excluded group. Second, the expelled students were also more likely to be found among those identified as SEN (38% vs only 17% among those who never experience a suspension or permanent exclusion and 33% among suspended). This group also had a large proportion of students whose parents had been contacted by the social services due to their children’s behaviour (26% compared to 2% never excluded and 10% suspended).

**Table 1:** Summary statistics on social background variables by school suspension/exclusion status

Socio‐demographic characteristics	Never excluded			Temp. suspended			Expelled		
	Mean (*SD*)	Min	Max	Mean (*SD*)	Min	Max	Mean (*SD*)	Min	Max
Girls	0.58 (0.49)	0	1	0.38 (0.49)	0	1	0.40 (0.49)	0	1
N. of siblings	1.57 (1.13)	0	9	1.71 (1.20)	0	9	1.95 (1.45)	0	8
N. of household members	4.47 (1.31)	1	14	4.48 (1.40)	2	12	4.62 (1.67)	2	12
SES	−0.23 (0.93)	−2	2	0.10 (0.95)	−2	2	0.51 (0.90)	−2	2
English not main language	0.10 (0.30)	0	1	0.06 (0.24)	0	1	0.12 (0.32)	0	1
Ethnicity: Black Caribbean	0.03 (0.17)	0	1	0.05 (0.23)	0	1	0.13 (0.34)	0	1
Mother age	41.74 (5.37)	18	68	40.48 (5.38)	22	57	40.07 (6.07)	23	64
Lone parent	0.19 (0.39)	0	1	0.32 (0.47)	0	1	0.47 (0.50)	0	1
IDACI	0.21 (0.17)	0	1	0.26 (0.19)	0	1	0.31 (0.16)	0	1
Resp. identified as SEN	0.17 (0.37)	0	1	0.33 (0.47)	0	1	0.38 (0.49)	0	1
Parents contacted by social services	0.02 (0.14)	0	1	0.10 (0.30)	0	1	0.26 (0.44)	0	1
Observations	5,835			711			86		

Note

SES score = positive values indicate the most deprived families; IDACI score = positive values indicate less economic deprived areas.

### Regression analyses

**Figure 1:**
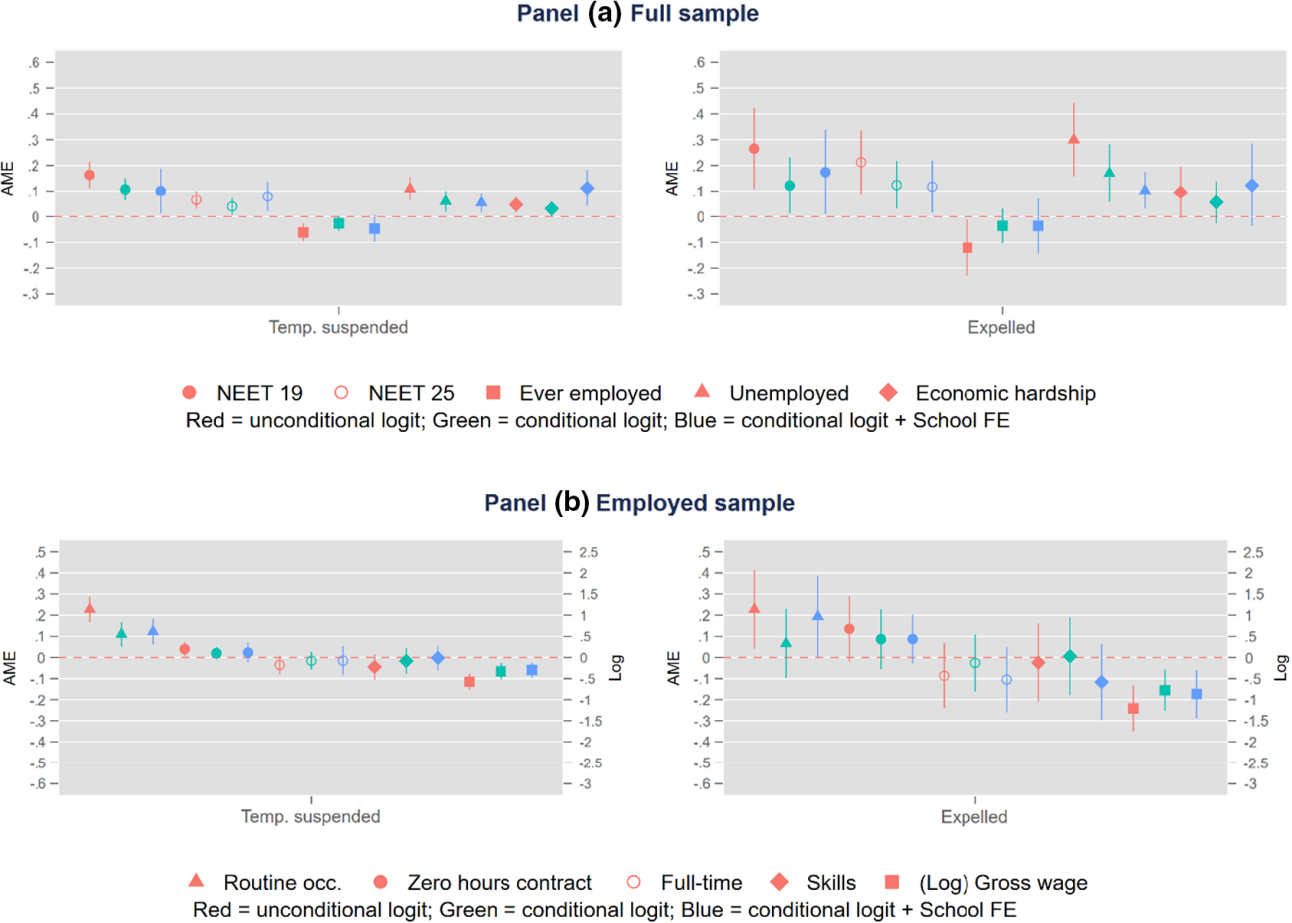
Average Marginal Effects (AME) on labour market and economic outcomes. *Note:* three different model specifications were used a) unconditional logit; b) conditional logit c) conditional logit with school fixed effects. LYPSE data. Adjusted standard errors at school level. Confidence intervals at 95% level. Attrition and nonresponse weights were used. The left‐side in the Y‐axis is a probability scale while in the right‐side is the Log‐transformation for the income variable.

Figure 1, panel a, displays the Average Marginal Effects (AME), that is, discrete changes in the probability scale, on the risk of being NEET at age 19/20 and 25/26, past employment experiences, risk of unemployment, and experiencing economic hardship also at age 25/26, while panel b displays the employment conditions of those who reported to have a job among suspended and expelled students (the reference category is never excluded).^5^ Red markers represent the differences in means, obtained from the unconditional regressions, between the groups of respondents in consideration and the reference category (i.e., never excluded). Green markers are the AME for the conditional regressions while blue markers are instead those AME from school‐fixed effects models (including all the controls for the social background used in the prior model). The point estimates tended to be quite close between them across the three model specifications, with standard errors becoming larger with the inclusion of control variables. Taken together, results point to the conclusion that both suspended and expelled students have experienced several disadvantages in early adulthood, even among those who find employment. More specifically, based on the school‐fixed effects specification, respondents who experienced suspension and permanent exclusions from school are had a higher risk of being NEET at age 19/20 (0.10 for suspended and 0.17 for expelled) and of remaining NEET later on at age 25/26 (0.08 and 0.12, respectively) or unemployed as well (0.05 and 0.10, respectively). They are also likely to have fewer employment experiences and suffer more often economic hardship, but none of these results are statistically significant once their socio‐demographic characteristics are accounted for. In terms of disadvantaged in employment conditions, we note from the last model specification that both groups report lower gross wages (−0.30 for temporarily suspended and −0.87 for expelled). Furthermore, these findings also suggest that permanent exclusions, in comparison to suspensions, are associated with worse outcomes.

### Counterfactual analyses

**Figure 2:**
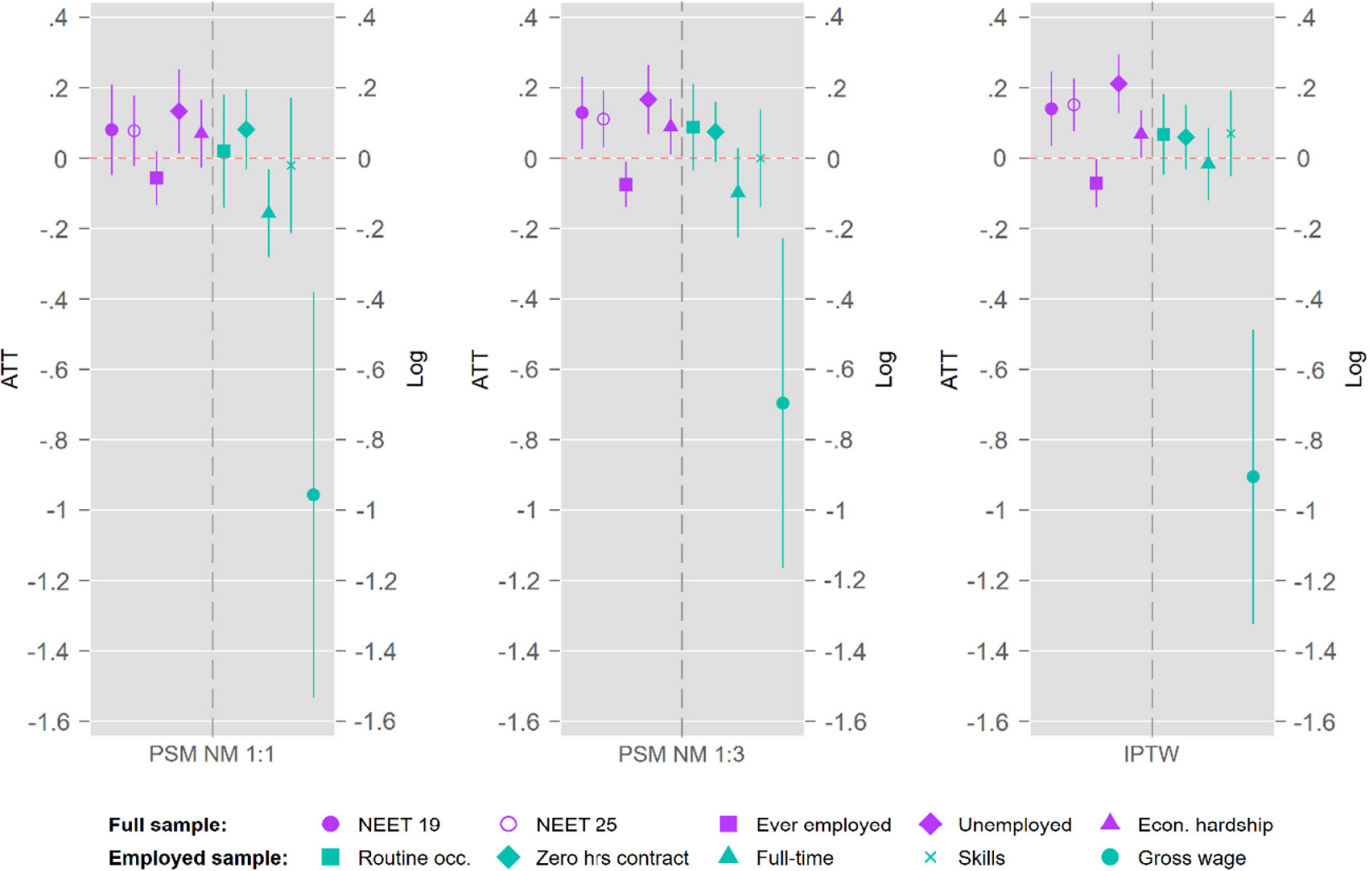
Results from PSM and IPTW. Average Treatment Effects on Treated (ATT) on labour market and economic outcomes. Expelled students. *Note: D* = 1 ‘Expelled’ vs *D* = 0 ‘Never excluded’; PSM NM 1:1 = Propensity Score Matching (nearest neighbour matching), one‐to‐one match; PSM NM 1:3 =Propensity Score Matching (nearest neighbour matching), one‐to‐three match; IPTW = Inverse Probability Treatment Weighting. Confidence intervals at 95% level. The left‐side in the Y‐axis is a probability scale while in the right‐side is the Log‐transformation for the income variable.

Following successful matching, we tested three different counterfactual models (*see* Appendix S1, pages 8–10, for a detailed description of the matching procedure). Figure 2 displays the Average Treatment Effects on Treated (ATT) across three different model specifications: (a) the PSM with one‐to‐one matching, (b) PSM with one‐to‐three matching, and (c) the IPTW. In these analyses, the suspended respondents were removed from the analysis, resulting in a dichotomous treatment variable in which *D* = 1 are the expelled respondents (*N* = 86) and *D* = 0 those never temporarily or expelled (*N* = 6,632). As before, full results are reported in Appendix S1 (Tables S3 and S4).

Our counterfactual models confirm the majority of the findings based on regression analyses presented in Figure 1. However, point estimates from the counterfactual models were higher than those obtained by standard regression adjustment, most likely because the regression adjustment was downwardly biased due to sample selection bias (i.e., more disadvantaged respondents with higher probability of school exclusion and poorer outcomes are likely to dropout). Further, although PSM and IPTW showed very similar point estimates, efficiency was gained by exploiting all the data available (*see* PSM NM 1:3 and IPTW panels). In particular, by looking at the IPTW estimator, we observed that the ATT for having previous employment experiences was −0.07, the risk of unemployed was 0.21, being NEET at age 19/20 was 0.14 and inactive at age 25/26 was 0.15 (all these estimates with a level of significance between 99% and 95% of confidence). We also observed an ATT of 0.07 for the risk of experiencing economic hardship but this was only significant at 90%. Among those employed, we also found a lower ATT for the (log) gross monthly wages, −0.91 (at a 99% level), a higher ATT for routine occupations (0.07), zero‐hours contract (0.06), and lower ATT for being in a full‐job (−0.02) but none of these estimates was statistically different from 0 as shown by the overlapping confidence intervals. In other words, even those who found a job still experienced several disadvantages in the labour market. However, we may have not detected any significant effect in these outcomes due to potential power issues since the sample of those employed was considerably smaller.

### Sensitivity analyses

Finally, we conducted a series of sensitivity analysis to assess how robust our results are to potential unobserved or omitted variables. We performed the Mantel–Haenszel bounds for our nonlinear outcomes (Becker & Caliendo, 2007) and the Rosembaum bounds (DiPrete & Gangl, 2004) for the earnings outcome. For the sake of space, we present the full tables in Appendix S1 and briefly comment on our results here. For the NEET outcomes, we found that potential hidden bias should be between 1.5 and 1.45 times higher to negate the results. For unemployment risk, the hidden bias should be above 1.5 times higher. We did not find enough evidence to reject the possibility of adjusting for potential bias nullifying the effect for the ever‐employed variable (although the bounds show a higher risk of underestimating this effect) and economic hardship (hidden bias should be 1.05 higher). In regard to the employed sample, we found that to wash away the effects on the probability of having a precarious employment contract and wages the hidden bias should be 1.35 and above 1.5, respectively while we did not find, again, enough evidence to reject confounding in the other employment outcomes (i.e., routine occupations, full‐time job, and job requires skills). However, the effects on these outcomes were also not statistically significant in both PSM and IPTW analyses. These null effects, however, could be due to the limited nature of our sample sizes and, in more substantial terms, they did not change any of our conclusions since we observed both several significant disadvantages in the transition to employment and in some of the qualitative aspects of the job attained (i.e., contract and wages).

In summary, our counterfactual analyses have shown that school exclusions have pervasive negative effects over the life course and can, unintentionally, exacerbate inequality and social exclusion in society through unemployment. Specifically, we found that school exclusion increases the risk of being NEET at the age 19 and, then, remaining NEET (economically inactive) and suffering more often unemployment at the age of 25. Further, these disadvantages do not end there but they are also present once these people start to work. Excluded respondents at the age of 25 are also at risk of remaining trapped in precarious jobs (with zero‐hours contracts) and earn much lower wages than similar counterparts that only differ because they have not been excluded from school.

## Discussion

Previous research has indicated that school exclusion can lead to worse outcomes for exposed youth; however, this evidence is based on short‐term outcomes or/and correlational analyses that have not – to date – necessarily provided the robust evidence needed to understand which outcomes are affected and inform policies to mitigate its adverse impacts. The purpose of this study was, therefore, to apply complementary counterfactual methods to a high‐quality UK longitudinal study to help illuminate the impact of school exclusion on long‐term labour market and economic outcomes. Results suggested that school exclusion during early adolescence increases the risk of a young person being NEET at age 18/19, confirming past findings on this outcome. However, we also found that school exclusion increased the risk of being unemployed, economically inactive, and earning less at age 25/26. Our study thus shows that the negative effects of school exclusion seem to be pervasive. Even those excluded respondents who manage to find a job still tended to experience large disadvantages in the labour market during early adulthood as denoted by their lower earnings.

Importantly, our findings suggested that our results were robust to school fixed specification. That is, potential endogeneity from unobserved school factors and self‐selection into schools is not compromising our conclusions since there are substantial effects of the exclusions even within schools. In other words, suspension or permanent exclusions have employment consequences no matter what school a young person had been excluded from. In fact, past research (Perry & Morris, 2014) suggests the reverse effects; whereby school exclusions have an adverse effect on academic achievement of non‐excluded students and overall performance of schools.

School exclusions, therefore, represent a precursor to exclusion from society in adulthood. There are likely to be multiple interacting mechanisms through which school exclusion could lead to poorer labour market and economic outcomes. For example, excluded students are estranged from the school environment in which fundamental aspects of social life are acquired and academic learning time is lost, resulting in lower socioemotional and cognitive skills and educational credentials. These processes will, in conjunction, negatively affect the transition of youth into adulthood and increase the risk of marginalization from society. In particular, boys from disadvantaged socioeconomic background, non‐intact families, and ethnic minorities such as black Caribbean are more at risk of being excluded from school (Strand & Fletcher, 2014). These factors have been largely documented to be associated with lower socioeconomic status in adulthood. Thus, permanent exclusion from school may largely aggravate educational and life course inequality in vulnerable groups.

Notably, our findings are consistent with findings presented in correlational studies related to suspension and permanent exclusion from school (*see* e.g., Massey, 2011; Spielhofer et al., 2009) as well as the unpublished Sutherland and Eisner (2014) study that used a similar matching method to ours to explore the impact of suspension on NEET at age 19. These findings together suggest that the impact of suspensions may be similar to those of permanent exclusions. However, future studies, based on more information related to the duration of suspensions than was available to us and to Sutherland and Eisner (2014), need to be carried out to answer questions related to the impact of suspensions of specific lengths. Arguably an exclusion of any length may impact on young people’s sense of fairness, belonging to school and being heard/seen by those who are in roles of authority (Obsuth et al., 2017). This may in turn influence their school engagement, attainment, and later employment. These and other potential mechanisms linking exclusions to later unemployment outcomes will also need to be examined by future research. Insights into these processes may facilitate the educational sector’s representatives’ (from policy makers to individual school representatives) understanding of the detrimental impact of school exclusion and lead to change in disciplinary practices. These authors maintain that, much like in the justice system, what is needed is a move from punishment through exclusion to rehabilitation through inclusion.

While in this study our focus was on the individual‐level consequences of school exclusion, the effects we identified are likely to have society‐level costs too; societal costs that are often not discussed in the policy agenda. First, from a pure economic perspective, a loss of human capital reduces productivity and economic growth in society. For example, higher rates of unemployment and economic inactivity increase welfare dependency and deteriorates health and well‐being. Second, by leaving behind vulnerable groups it also increases social inequality and undermines the idea of justice and equality of opportunity in society (Thompson, 2020).

### Strengths and limitations

Using rich longitudinal data and counterfactual methods, this study provided robust evidence of the consequence of school exclusion on mid‐to‐long‐term employment and economic outcomes in England. However, there are four major limitations in this study that could be addressed in future research. First, this study focused on comparing never excluded versus temporarily suspended and expelled students, but we were not able to distinguish between early and late exclusions. Exclusion events during primary education rather than in secondary education might have different consequences in pupil educational and life trajectories. Second, we also lacked disaggregated information on the length, number of exclusions, and the reasons for permanently excluding students from school. Third, information on school exclusions were self‐reported by parents which might lead to measurement error issues. If this is the case, our estimates are attenuated, and the effects of the exposure variable should be larger. This, however, should not change our conclusions. Fourth, our sample size was relatively small to further investigate potential heterogeneities by gender and ethnicity. All these factors are important for understanding the mechanisms that lead into exclusions and can provide crucial information for designing targeted interventions. Linked governmental micro data on school exclusions, education, employment, and justice should enable to explore these issues and improve our understanding regarding the predictors and consequences of school exclusions.

### Conclusions

Our findings suggest that school exclusion has a negative impact on the labour market outcomes of exposed youth. Though other studies have identified such an association previously, our study provides some of the most robust evidence to date. Given the evidence of the harms of school exclusion, policy makers should look at implementing both, early interventions for preventing school exclusion but also interventions aimed at mitigating the negative consequences of school exclusion and re‐integrating excluded youth. For instance, through life‐long learning programmes the risk of becoming NEET could potentially be reduced, provide school excluded individuals with competences and qualifications that can smooth their transition into the labour market and make them fully active members of society.

## Conflicts of interest

All authors declare no conflict of interest.

## Supporting information


**Appendix S1**. Materials.

## Data Availability

The data that support the findings of this study are openly available in the UK Data Service at https://beta.ukdataservice.ac.uk/datacatalogue/studies/study?id=5545, reference number [SN5545].

## Footnotes

^1^
After the selection of respondents present in the final wave, there were no excluded students in independent schools (i.e., private institutions). Since our data might not be good enough in representing this very selected group we decided to focus only on respondent from maintained schools (i.e., state funded schools).

^2^
We also employed a 1:1 nearest neighbour matching without replacement using Becker and Ichino (2002) procedure, obtaining similar point estimates but less efficient standard errors.

^3^
In our case, IPTW is good strategy to tackle potential endogeneity from selection bias. Our marginal structural model first accounts for the selection into the treatment and then uses the estimated PS to achieve balance between control and treatments groups without discarding any observation in the sample.

^4^
For a comparison of the sample before‐after attrition see table A.1 in the appendix section.

^5^
All the regression coefficients from these models are available in the Supplemental material (Tables S.2.a, b and c).
