# Supplementary material for: Long‐term labour market and economic consequences of school exclusions in England: Evidence from two counterfactual approaches
Source: Br J Educ Psychol. 2022 Feb 9;92(3):801–16. doi: 10.1111/bjep.12487 (PMC9546012; doi:10.1111/bjep.12487)
Supplement: Supplementary file 1 — Appendix S1. Materials. [file BJEP-92-801-s001.docx]

## **Supplemental materials**

***Sample attrition and selection on maintained schools***

Another interesting aspect of our data is the sample attrition which might indirectly represent some of the disadvantaged experienced during adolescence and early adulthood for those students who were permanently excluded from school. Figure A.1 is a simplified description of the sample attrition and percentage of individual who transited from school to employment or education.

S.1: Description of attrition in LYPSE/Next Steps Survey


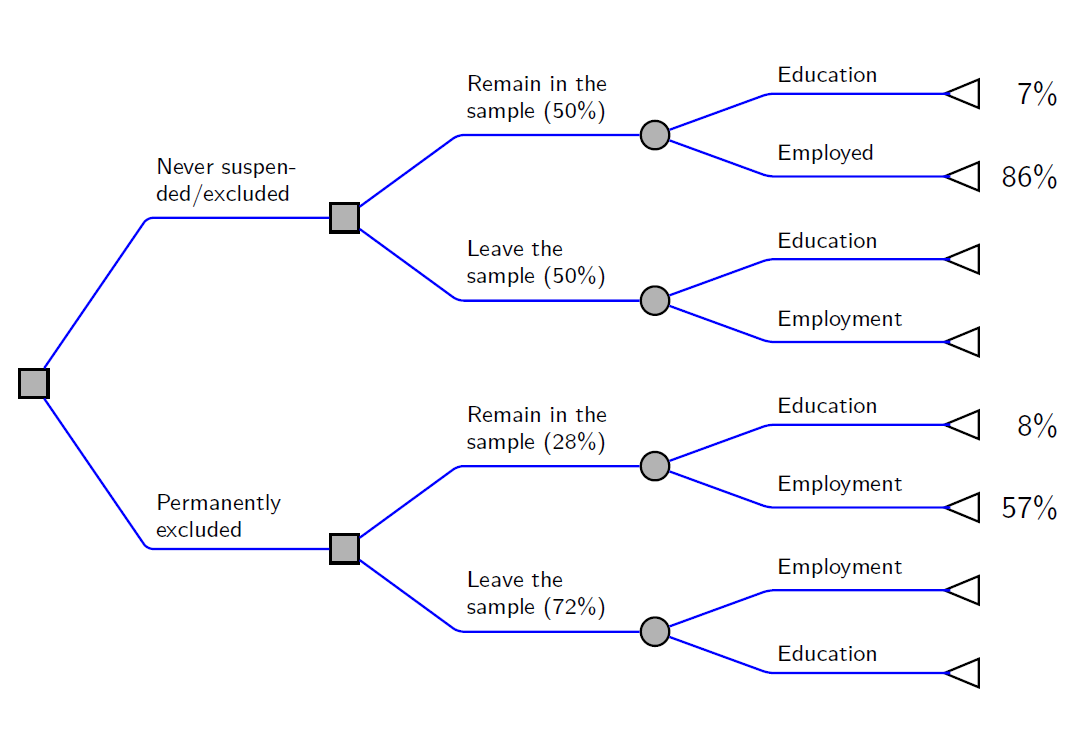


Sample attrition is a very common issue in panel data. However, in our study we can also observe a disproportion of sample dropouts among those students who expelled from school. Taking a deeper look into the reasons why expelled students have not participated into the latest LYPSE study (see figure A.2), we found that the 50% of the non-participants were not surveyed due to the impossibility of tracing and contacting them. If we compare this pattern between never excluded and expelled students, we see that there is almost a 25-percentage points difference. This is exactly the double than those who never experienced a temporary suspension or expulsion. In more substantive terms, these different patterns might indicate, as part of a cumulative disadvantage, that some of these respondents do not have a permanent home or could have even become homeless. In other words, our sample might reflect the outcomes for those who have not experienced the worse consequences of school exclusion.

S.2: Classification of non-participants in wave 8 of LYPSE/Next Steps Survey

Table S.1: Classification of non-participants in wave 8 of LYPSE/Next Steps Survey

|  | Initial sample | | | | After attrition | | | | After listwise deletion of missingness | | | |
| --- | --- | --- | --- | --- | --- | --- | --- | --- | --- | --- | --- | --- |
|  | mean | sd | min | max | mean | sd | min | max | mean | sd | min | max |
| School exclusion |  |  |  |  |  |  |  |  |  |  |  |  |
| *Never excluded* | 0.84 | (0.36) | 0 | 1 | 0.89 | (0.32) | 0 | 1 | 0.88 | (0.33) | 0 | 1 |
| *Temp. suspended* | 0.13 | (0.34) | 0 | 1 | 0.10 | (0.30) | 0 | 1 | 0.11 | (0.31) | 0 | 1 |
| *Expelled* | 0.02 | (0.15) | 0 | 1 | 0.01 | (0.11) | 0 | 1 | 0.01 | (0.11) | 0 | 1 |
| Socio-demographic characteristics |  |  |  |  |  |  |  |  |  |  |  |  |
| *Girls* | 0.49 | (0.50) | 0 | 1 | 0.56 | (0.50) | 0 | 1 | 0.55 | (0.50) | 0 | 1 |
| *N. of siblings* | 1.65 | (1.26) | 0 | 11 | 1.62 | (1.20) | 0 | 9 | 1.59 | (1.15) | 0 | 9 |
| *N. of household members* | 4.50 | (1.45) | 1 | 15 | 4.50 | (1.41) | 1 | 14 | 4.48 | (1.33) | 1 | 14 |
| *Family SES* | -0.00 | (1.00) | -2 | 2 | -0.16 | (0.97) | -2 | 2 | -0.19 | (0.94) | -2 | 2 |
| *English is not main language* | 0.13 | (0.34) | 0 | 1 | 0.12 | (0.33) | 0 | 1 | 0.09 | (0.29) | 0 | 1 |
| *Ethnicity: Black Caribbean* | 0.05 | (0.21) | 0 | 1 | 0.03 | (0.18) | 0 | 1 | 0.03 | (0.18) | 0 | 1 |
| *Mother age* | 41.32 | (5.64) | 18 | 74 | 41.77 | (5.54) | 18 | 74 | 41.59 | (5.39) | 18 | 68 |
| *Grew with Lone parent* | 0.26 | (0.44) | 0 | 1 | 0.22 | (0.41) | 0 | 1 | 0.21 | (0.41) | 0 | 1 |
| *IDACI* | 0.24 | (0.18) | 0 | 1 | 0.22 | (0.18) | 0 | 1 | 0.21 | (0.17) | 0 | 1 |
| *Identified as having special educational needs* | 0.21 | (0.40) | 0 | 1 | 0.19 | (0.39) | 0 | 1 | 0.19 | (0.39) | 0 | 1 |
| *Parents contacted by social services* | 0.04 | (0.21) | 0 | 1 | 0.03 | (0.18) | 0 | 1 | 0.03 | (0.17) | 0 | 1 |
| **Observations** | **16122** | | | | **7707** | | | | **6632** | | | |

Note: SES score = positive values indicate the most deprived families; IDACI score = positive values indicate less economically deprived areas

S.3: Percentage of temporary suspended and expelled students by type of school attended

******

There were no permanently excluded cases among those who attended independent schools (they represent the 3.8% of the sample). This could be the case because independent schools are a very selected group, and our sample might not be good enough in representing this population. For this reason, we also decided to drop these observations from the final sample.

***Standard logistic and OLS regression models***

Table S.2.a: AME from unconditional nonlinear regressions

|  | **Full sample** | | | | | | | | | |
| --- | --- | --- | --- | --- | --- | --- | --- | --- | --- | --- |
|  | NEET 19-20 | | NEET 25-26 | | Ever employed | | Unemployed | | Eco hardship | |
| School exclusion (ref. never excluded) | AME | se | AME | se | AME | se | AME | se | AME | se |
| *Temp. suspended* | 0.163*** | (0.026) | 0.065*** | (0.018) | -0.062*** | (0.018) | 0.109*** | (0.023) | 0.046*** | (0.015) |
| *Expelled* | 0.265*** | (0.080) | 0.213*** | (0.063) | -0.121** | (0.055) | 0.300*** | (0.073) | 0.095* | (0.051) |
| **pseudo R2** | 0.051 |  | 0.019 |  | 0.016 |  | 0.021 |  | 0.009 |  |
| **N** | 5,327 |  | 6,632 |  | 6,632 |  | 6,632 |  | 6,632 |  |
|  | **Employed sample** | | | | | | | | | |
|  | Routine occ. | | Zero hours cont. | | Full-time | | Skills | | Log Wages | |
| School exclusion (ref. never excluded) | AME | se | AME | se | AME | se | AME | se | AME | se |
| *Temp. suspended* | 0.228*** | (0.031) | 0.040** | (0.017) | -0.035 | (0.022) | -0.045 | (0.031) | -0.574*** | (0.098) |
| *Expelled* | 0.227** | (0.095) | 0.136* | (0.079) | -0.086 | (0.079) | -0.024 | (0.095) | -1.205*** | (0.274) |
| **pseudo R2/R2** | 0.019 |  | 0.011 |  | 0.002 |  | 0.001 |  | 0.018 |  |
| **N** | 4,641 |  | 4,641 |  | 4,641 |  | 4,641 |  | 4,641 |  |
| Note: Standard errors in parentheses; * p < 0.10, ** p < 0.05, *** p < 0.01; Attrition weights were used and adj. std err. at school level; | | | | | | | | | | |

Table S.2.b: AME from conditional nonlinear regressions

|  | **Full sample** | | | | | | | | | |
| --- | --- | --- | --- | --- | --- | --- | --- | --- | --- | --- |
|  | NEET 19-20 | | NEET 25-26 | | Ever employed | | Unemployed | | Eco hardship | |
|  | AME | se | AME | se | AME | se | AME | se | AME | se |
| School exclusion (ref. never excluded) |  |  |  |  |  |  |  |  |  |  |
| *Temp. suspended* | 0.107*** | (0.022) | 0.039** | (0.017) | -0.027* | (0.015) | 0.059*** | (0.021) | 0.031** | (0.015) |
| *Expelled* | 0.122** | (0.056) | 0.124*** | (0.048) | -0.036 | (0.034) | 0.169*** | (0.057) | 0.056 | (0.042) |
| Covariates |  |  |  |  |  |  |  |  |  |  |
| *Woman* | 0.014 | (0.012) | 0.094*** | (0.011) | -0.044*** | (0.009) | 0.082*** | (0.012) | 0.015* | (0.009) |
| *N. Siblings* | -0.014 | (0.014) | 0.011 | (0.015) | -0.012 | (0.015) | -0.011 | (0.015) | -0.004 | (0.011) |
| *N. hh members* | 0.013 | (0.013) | -0.004 | (0.014) | 0.011 | (0.014) | 0.016 | (0.014) | 0.005 | (0.011) |
| *Family SES* | 0.054*** | (0.008) | 0.028*** | (0.006) | -0.034*** | (0.006) | 0.052*** | (0.007) | 0.017*** | (0.006) |
| *English not main language* | -0.107*** | (0.024) | -0.057*** | (0.020) | -0.032** | (0.015) | -0.004 | (0.023) | 0.000 | (0.017) |
| *Ethnicity: Black Caribbean* | -0.051* | (0.029) | -0.076*** | (0.029) | 0.043* | (0.024) | -0.078** | (0.039) | -0.010 | (0.020) |
| *Mother age* | 0.001 | (0.001) | -0.001 | (0.001) | -0.001 | (0.001) | -0.000 | (0.001) | 0.000 | (0.001) |
| *Lone parent* | 0.025 | (0.018) | -0.006 | (0.017) | 0.015 | (0.016) | 0.025 | (0.019) | 0.020 | (0.016) |
| *IDACI* | 0.063* | (0.037) | 0.094*** | (0.028) | -0.069** | (0.029) | 0.166*** | (0.037) | 0.012 | (0.027) |
| *Identified as SEN* | 0.021 | (0.013) | 0.057*** | (0.013) | -0.066*** | (0.011) | 0.086*** | (0.015) | 0.020** | (0.010) |
| *Parents contacted by social services* | 0.058** | (0.025) | 0.056*** | (0.020) | -0.059*** | (0.017) | 0.049* | (0.027) | 0.012 | (0.019) |
| **Pseudo R2** | 0.124 |  | 0.113 |  | 0.112 |  | 0.099 |  | 0.024 |  |
| **N** | 5,327 |  | 6,632 |  | 6,632 |  | 6,632 |  | 6,632 |  |
|  | **Employed sample** | | | | | | | | | |
|  | Routine occ. | | Zero hours cont. | | Full-time | | Skills | | Log Wages | |
|  | AME | se | AME | se | AME | se | AME | se | AME | se |
| School exclusion (ref. never excluded.) |  |  |  |  |  |  |  |  |  |  |
| Temp. *suspended* | 0.109*** | (0.030) | 0.020 | (0.015) | -0.016 | (0.021) | -0.016 | (0.032) | -0.328*** | (0.099) |
| Expelled | 0.066 | (0.084) | 0.086 | (0.072) | -0.026 | (0.068) | 0.006 | (0.094) | -0.775*** | (0.248) |
| Covariates |  |  |  |  |  |  |  |  |  |  |
| *Woman* | -0.094*** | (0.016) | -0.010 | (0.009) | -0.163*** | (0.014) | 0.007 | (0.018) | -0.399*** | (0.061) |
| *N. Siblings* | -0.001 | (0.024) | -0.007 | (0.011) | 0.020 | (0.016) | 0.015 | (0.027) | 0.036 | (0.092) |
| *N. hh members* | 0.018 | (0.022) | 0.010 | (0.011) | -0.024 | (0.016) | -0.013 | (0.025) | -0.078 | (0.090) |
| *Family SES* | 0.089*** | (0.010) | 0.017*** | (0.006) | -0.039*** | (0.008) | -0.045*** | (0.011) | -0.249*** | (0.037) |
| *English not main language* | -0.203*** | (0.042) | 0.010 | (0.019) | 0.015 | (0.028) | 0.117*** | (0.040) | 0.447*** | (0.138) |
| *Ethnicity: Black Caribbean* | 0.010 | (0.048) | 0.009 | (0.028) | 0.002 | (0.041) | 0.054 | (0.052) | 0.266* | (0.161) |
| *Mother age* | -0.002 | (0.002) | 0.001 | (0.001) | 0.003** | (0.001) | -0.001 | (0.002) | -0.001 | (0.006) |
| *Lone parent* | 0.013 | (0.030) | 0.015 | (0.014) | -0.043** | (0.022) | -0.011 | (0.032) | -0.117 | (0.120) |
| *IDACI* | 0.131** | (0.057) | 0.012 | (0.030) | -0.070* | (0.041) | -0.059 | (0.061) | -0.776*** | (0.190) |
| *Identified as SEN* | 0.153*** | (0.019) | 0.019* | (0.011) | -0.064*** | (0.016) | -0.023 | (0.023) | -0.617*** | (0.074) |
| *Parents contacted by social services* | 0.131** | (0.051) | 0.015 | (0.021) | -0.052 | (0.035) | -0.019 | (0.057) | -0.534*** | (0.203) |
| **Pseudo R2/R2** | 0.090 |  | 0.030 |  | 0.102 |  | 0.008 |  | 0.092 |  |
| **N** | 4,641 |  | 4,641 |  | 4,641 |  | 4,641 |  | 4,641 |  |
| Note: Standard errors in parentheses; * p < 0.10, ** p < 0.05, *** p < 0.01; Attrition weights were used and adj. std err. at school level. | | | | | | | | | | |

Table S.2.c: AME from nonlinear regressions with school fixed effects

|  | **Full sample** | | | | | | | | | |
| --- | --- | --- | --- | --- | --- | --- | --- | --- | --- | --- |
|  | NEET 19-20 | | NEET 25-26 | | Ever employed | | Unemployed | | Eco hardship | |
|  | AME | se | AME | se | AME | se | AME | se | AME | se |
| School exclusion (ref. never excluded.) |  |  |  |  |  |  |  |  |  |  |
| *Temp. suspended* | 0.109** | (0.047) | 0.074*** | (0.028) | -0.050* | (0.028) | 0.052*** | (0.019) | 0.112*** | (0.036) |
| *Expelled* | 0.180** | (0.085) | 0.121** | (0.052) | -0.054 | (0.058) | 0.102*** | (0.036) | 0.123 | (0.082) |
| Covariates |  |  |  |  |  |  |  |  |  |  |
| *Woman* | 0.005 | (0.022) | 0.198*** | (0.049) | -0.074*** | (0.027) | 0.092*** | (0.024) | 0.048* | (0.027) |
| *N. Siblings* | 0.002 | (0.025) | -0.001 | (0.020) | -0.015 | (0.025) | -0.011 | (0.012) | 0.017 | (0.033) |
| *N. hh members* | 0.017 | (0.020) | 0.018 | (0.017) | 0.002 | (0.021) | 0.018* | (0.010) | -0.019 | (0.031) |
| *Family SES* | 0.094*** | (0.033) | 0.043*** | (0.014) | -0.048*** | (0.017) | 0.046*** | (0.013) | 0.034** | (0.015) |
| *English not main language* | -0.055 | (0.051) | -0.056 | (0.036) | -0.049 | (0.037) | -0.004 | (0.021) | -0.040 | (0.052) |
| *Ethnicity: Black Caribbean* | 0.019 | (0.069) | -0.000 | (0.053) | -0.002 | (0.056) | -0.044 | (0.036) | -0.001 | (0.065) |
| *Mother age* | 0.002 | (0.001) | -0.001 | (0.002) | -0.002 | (0.001) | 0.001 | (0.001) | -0.000 | (0.002) |
| *Lone parent* | 0.048* | (0.029) | 0.038 | (0.025) | 0.000 | (0.032) | 0.042** | (0.017) | 0.018 | (0.041) |
| *IDACI* | 0.017 | (0.082) | 0.173** | (0.073) | -0.117 | (0.073) | 0.138*** | (0.051) | 0.128 | (0.102) |
| *Identified as SEN* | 0.095** | (0.039) | 0.107*** | (0.032) | -0.152*** | (0.047) | 0.098*** | (0.027) | 0.090*** | (0.030) |
| *Parents contacted by social services* | 0.144** | (0.065) | 0.066 | (0.042) | -0.119** | (0.051) | 0.045 | (0.030) | 0.070 | (0.060) |
| **Pseudo R2** | 0.138 |  | 0.110 |  | 0.071 |  | 0.074 |  | 0.025 |  |
| **N** | 2,462 |  | 3,591 |  | 3,460 |  | 5,177 |  | 3,614 |  |
|  | **Employed sample** | | | | | | | | | |
|  | Routine occ. | | Zero hours cont. | | Full-time | | Skills | | Log Wages | |
|  | AME | se | AME | se | AME | se | AME | se | AME | se |
| School exclusion (ref. never excluded) |  |  |  |  |  |  |  |  |  |  |
| *Temp. suspended* | 0.087*** | (0.026) | 0.057 | (0.035) | -0.021 | (0.022) | 0.023 | (0.026) | -0.308*** | (0.091) |
| *Expelled* | 0.170** | (0.083) | 0.147** | (0.075) | -0.103** | (0.049) | -0.016 | (0.078) | -0.770*** | (0.290) |
| Covariates |  |  |  |  |  |  |  |  |  |  |
| *Woman* | -0.074*** | (0.016) | -0.015 | (0.026) | -0.168*** | (0.037) | 0.020 | (0.016) | -0.361*** | (0.054) |
| *N. Siblings* | 0.029 | (0.021) | -0.000 | (0.027) | 0.012 | (0.017) | -0.008 | (0.019) | -0.080 | (0.068) |
| *N. hh members* | -0.016 | (0.020) | 0.019 | (0.022) | -0.019 | (0.014) | 0.011 | (0.018) | 0.025 | (0.063) |
| *Family SES* | 0.080*** | (0.011) | 0.030* | (0.017) | -0.023** | (0.010) | -0.036*** | (0.009) | -0.180*** | (0.033) |
| *English not main language* | -0.093*** | (0.036) | 0.050 | (0.048) | 0.008 | (0.028) | 0.057* | (0.032) | 0.085 | (0.113) |
| *Ethnicity: Black Caribbean* | 0.081* | (0.048) | 0.001 | (0.068) | -0.043 | (0.038) | 0.007 | (0.045) | -0.095 | (0.162) |
| *Mother age* | -0.002 | (0.001) | 0.002 | (0.002) | 0.000 | (0.001) | -0.003* | (0.001) | -0.011** | (0.005) |
| *Lone parent* | 0.016 | (0.029) | 0.069** | (0.035) | -0.067*** | (0.021) | 0.006 | (0.026) | -0.031 | (0.091) |
| *IDACI* | 0.052 | (0.065) | 0.005 | (0.093) | -0.107* | (0.057) | -0.015 | (0.061) | -0.498** | (0.215) |
| *Identified as SEN* | 0.146*** | (0.022) | 0.090** | (0.039) | -0.109*** | (0.029) | -0.010 | (0.020) | -0.592*** | (0.070) |
| *Parents contacted by social services* | 0.125*** | (0.047) | 0.096 | (0.066) | -0.078* | (0.041) | -0.100** | (0.048) | -0.522*** | (0.171) |
| **Pseudo R2/R2** | 0.065 |  | 0.035 |  | 0.066 |  | 0.006 |  | 0.051 |  |
| **N** | 4,978 |  | 2,378 |  | 4,496 |  | 5,417 |  | 4,803 |  |

Note: Standard errors in parentheses; * p < 0.10, ** p < 0.05, *** p < 0.01; Attrition weights were used and adj. std err. at school level. Variation within school clusters only.

Note that NEET 19 (wave 7) has a lower N than the rest of the outcomes. This is because at the age 25-26 (wave 8) all participants of the first wave were recontacted including those who not participated to wave 7 (age 19-20).

***Propensity Scores estimation***

| Table S.3: T-test on covariates after PS estimation | | | | | |
| --- | --- | --- | --- | --- | --- |
| Variables | **Mean** | | | **T-test** | |
|  | Treated | Control | %bias | t | p>\|t\| |
| SEN (sen) | 0.378 | 0.411 | -7.7 | -0.46 | 0.649 |
| Parents contacted by social services (socser2) | 0.256 | 0.200 | 17.1 | 0.89 | 0.377 |
| Gender (female) | 0.400 | 0.433 | -6.8 | -0.45 | 0.652 |
| N. siblings (sib) | 1.967 | 1.911 | 4.3 | 0.27 | 0.788 |
| N. household members (hhno) | 4.644 | 4.533 | 7.3 | 0.46 | 0.647 |
| SES score (ses3) | 0.693 | 0.716 | -2.5 | -0.16 | 0.872 |
| English not first language (lang) | 0.122 | 0.089 | 10.6 | 0.72 | 0.470 |
| Black Caribbean (ethni3) | 0.122 | 0.133 | -4.3 | -0.22 | 0.825 |
| Mother Age (mumage) | 40.156 | 38.733 | 24.7 | 1.6 | 0.111 |
| Lone parent (lonepar) | 0.467 | 0.567 | -22.2 | -1.34 | 0.181 |
| IDACE score (idaci2) | 0.318 | 0.279 | 23.2 | 1.53 | 0.129 |

Figure S.4: Standardized bias before/after PS estimation from table A.2

Note: The standardised percentage bias is the percentage difference of the sample means in the treated and non-treated (full or matched) sub-samples as a percentage of the square root of the average of the sample variances in the treated and non-treated groups (formulae from Rosenbaum and Rubin, 1985)

Figure S.5: Distribution of Propensity Scores before/after matching – All respondents


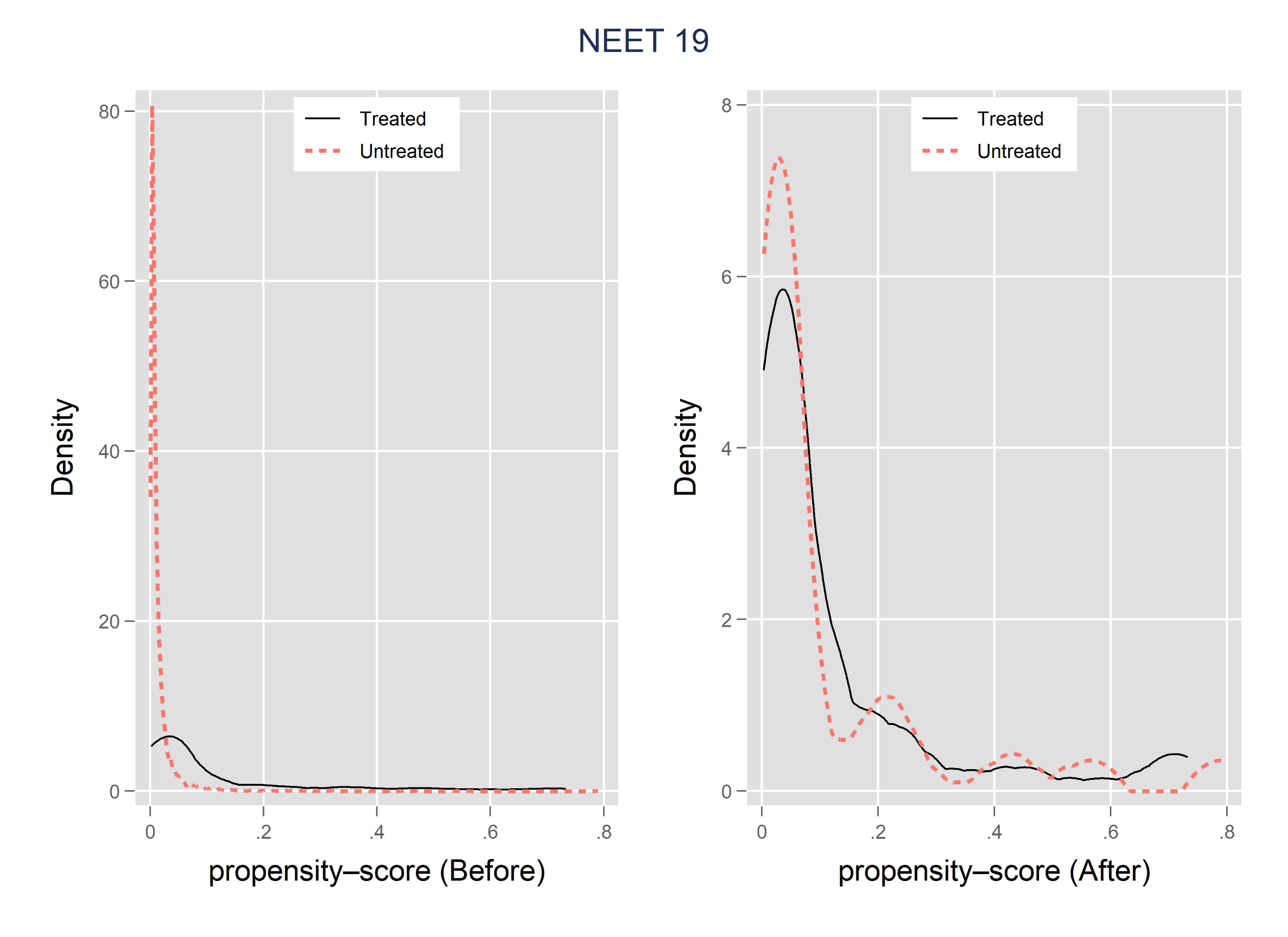

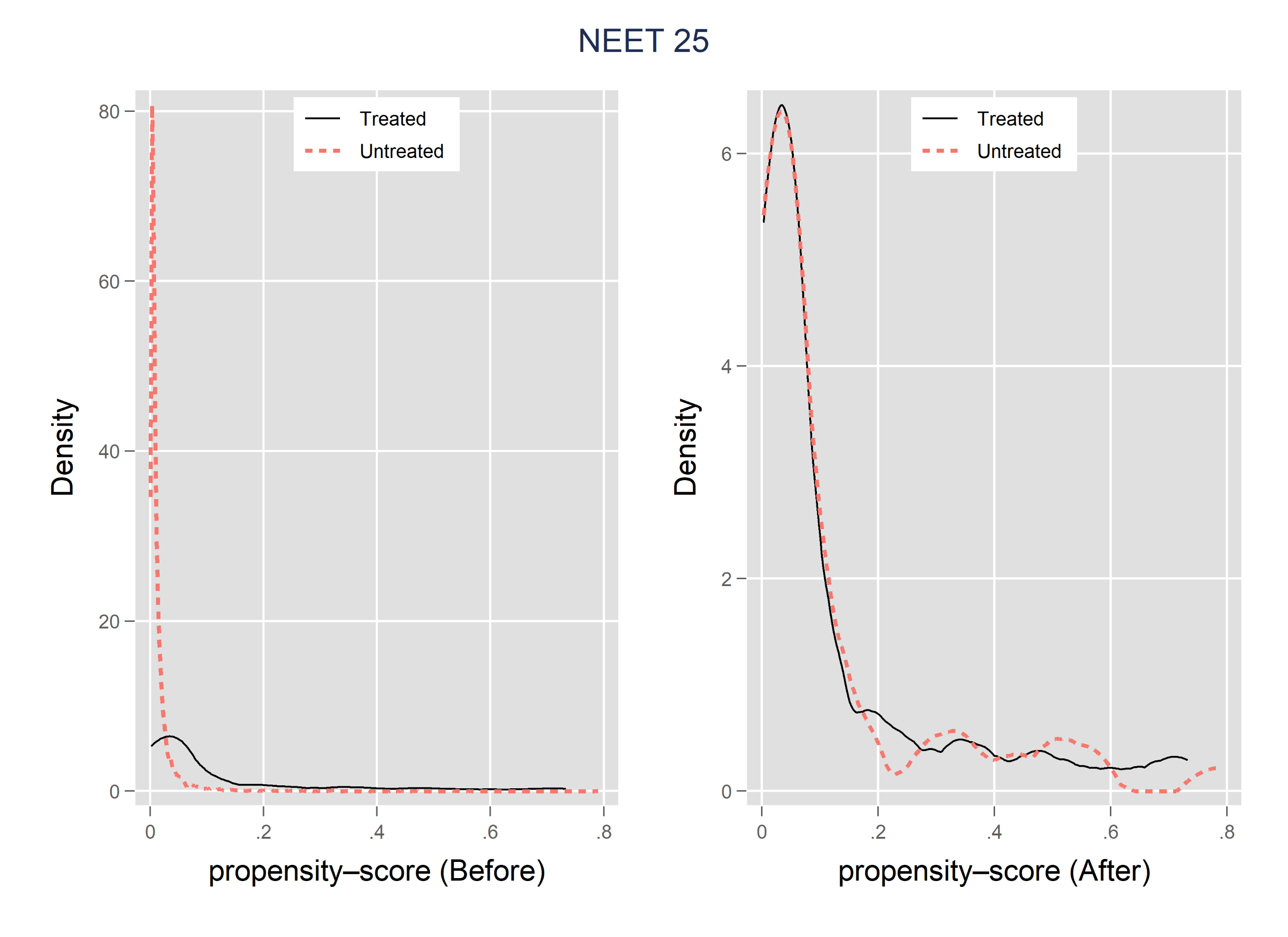


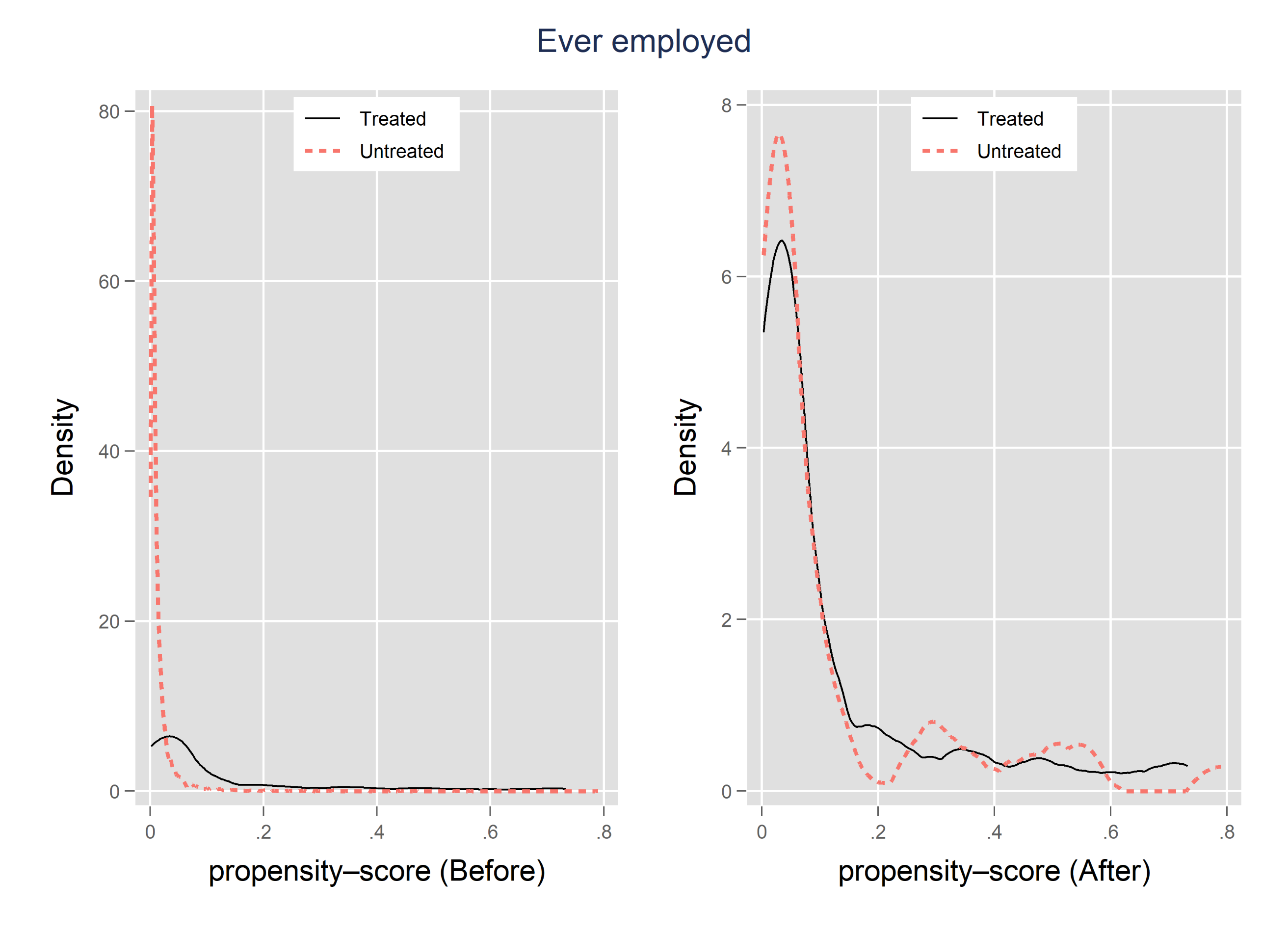

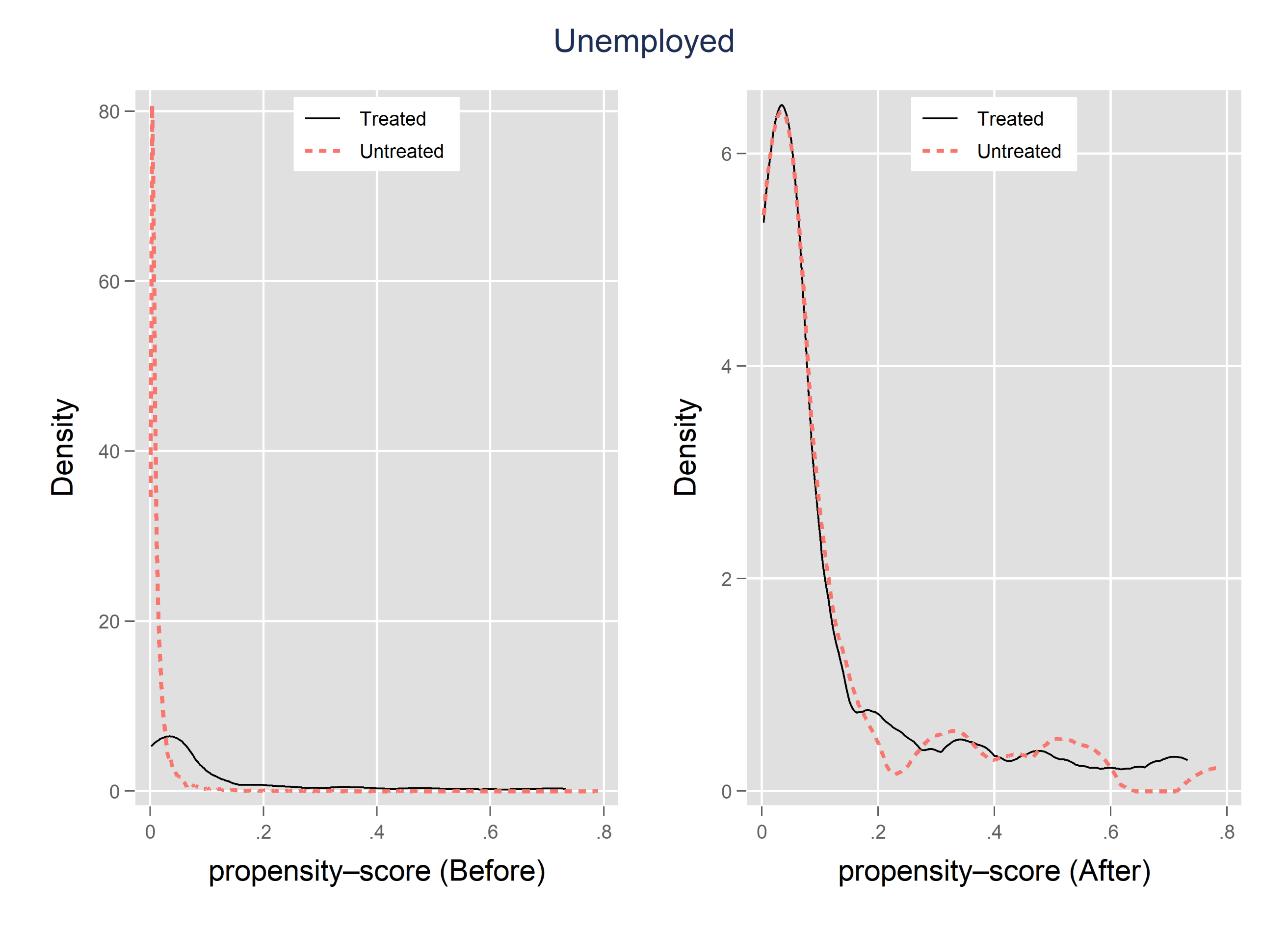

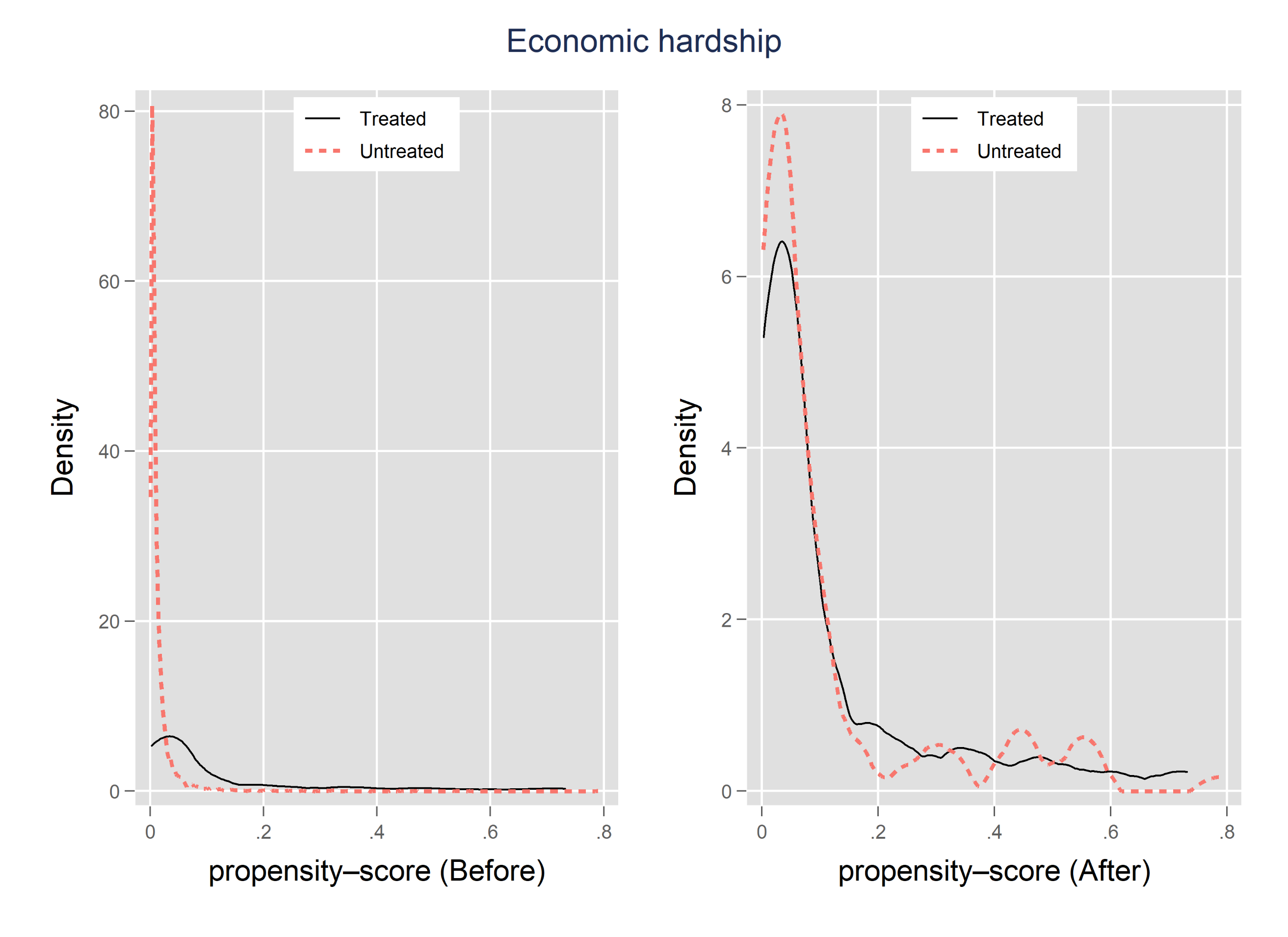


Figure S.6: Distribution of Propensity Scores before/after matching – Employed respondents


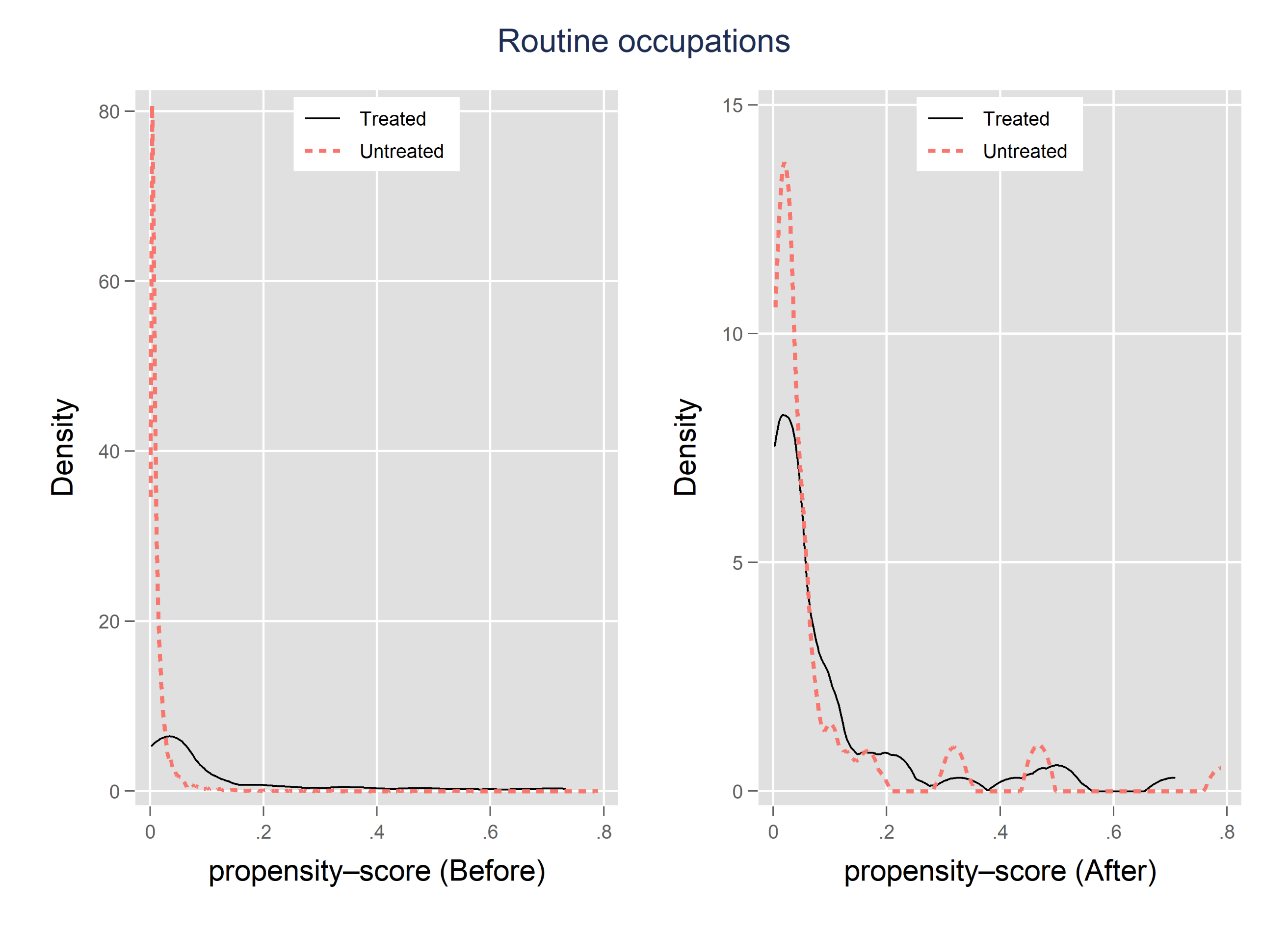

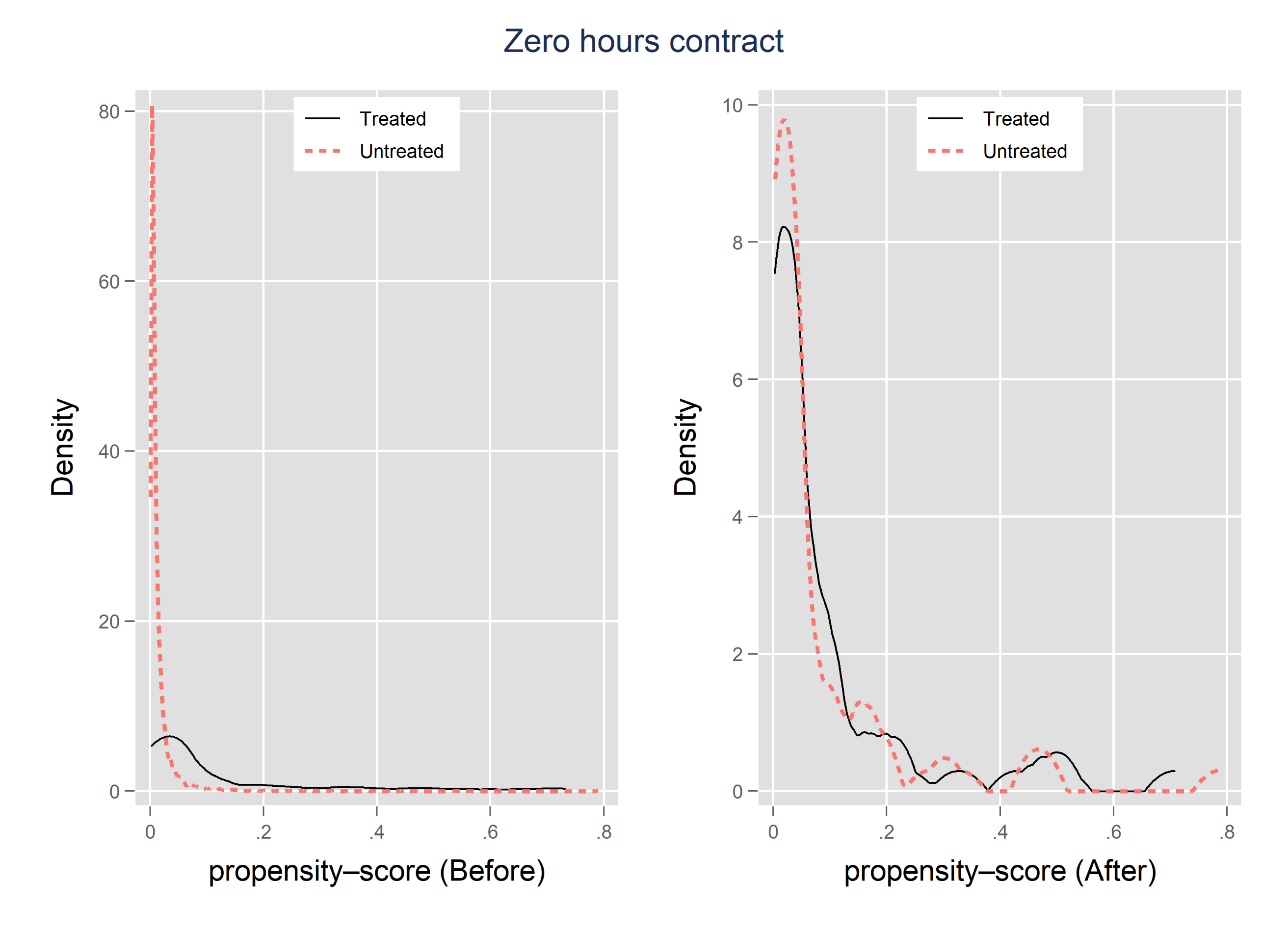


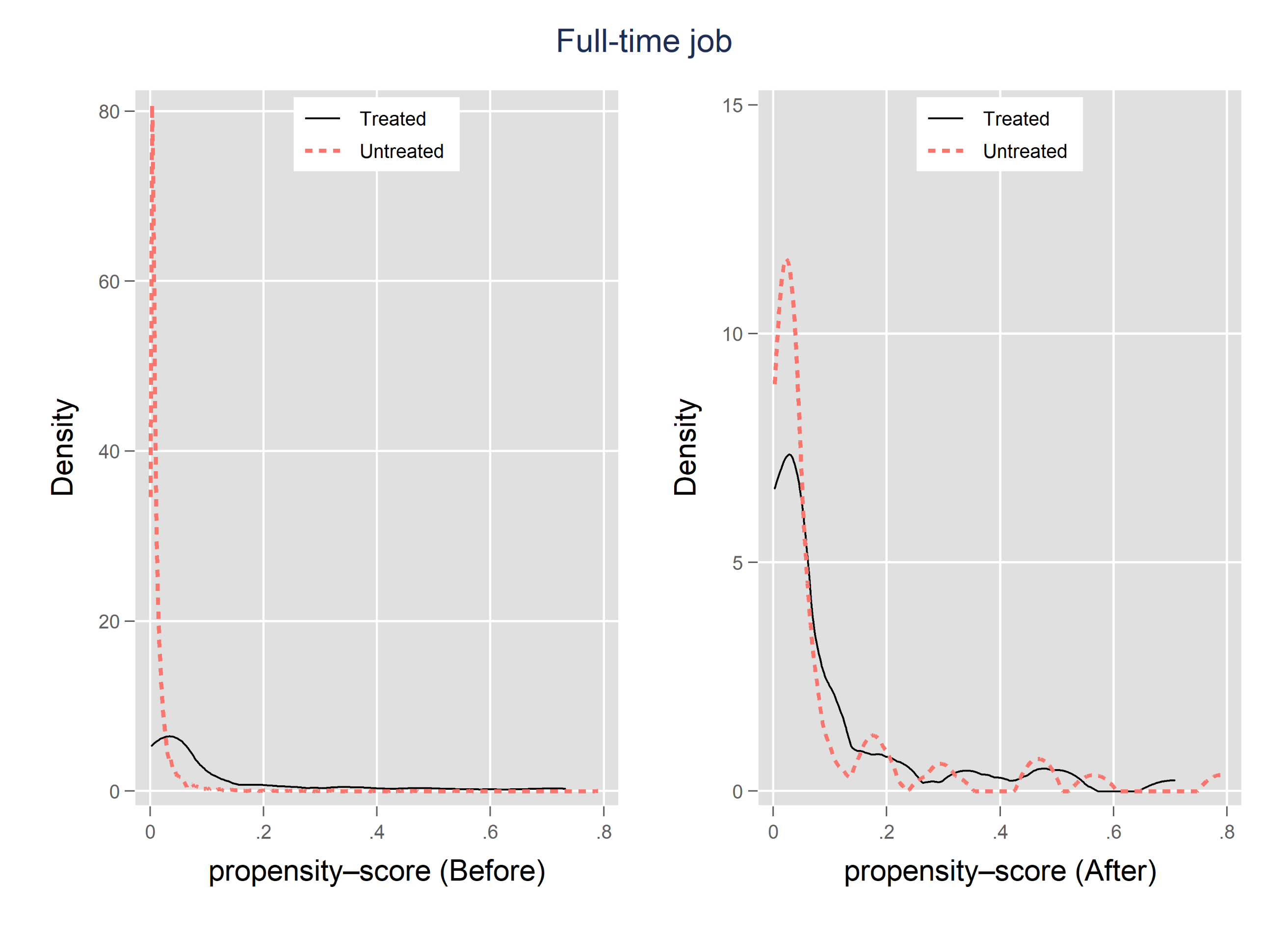

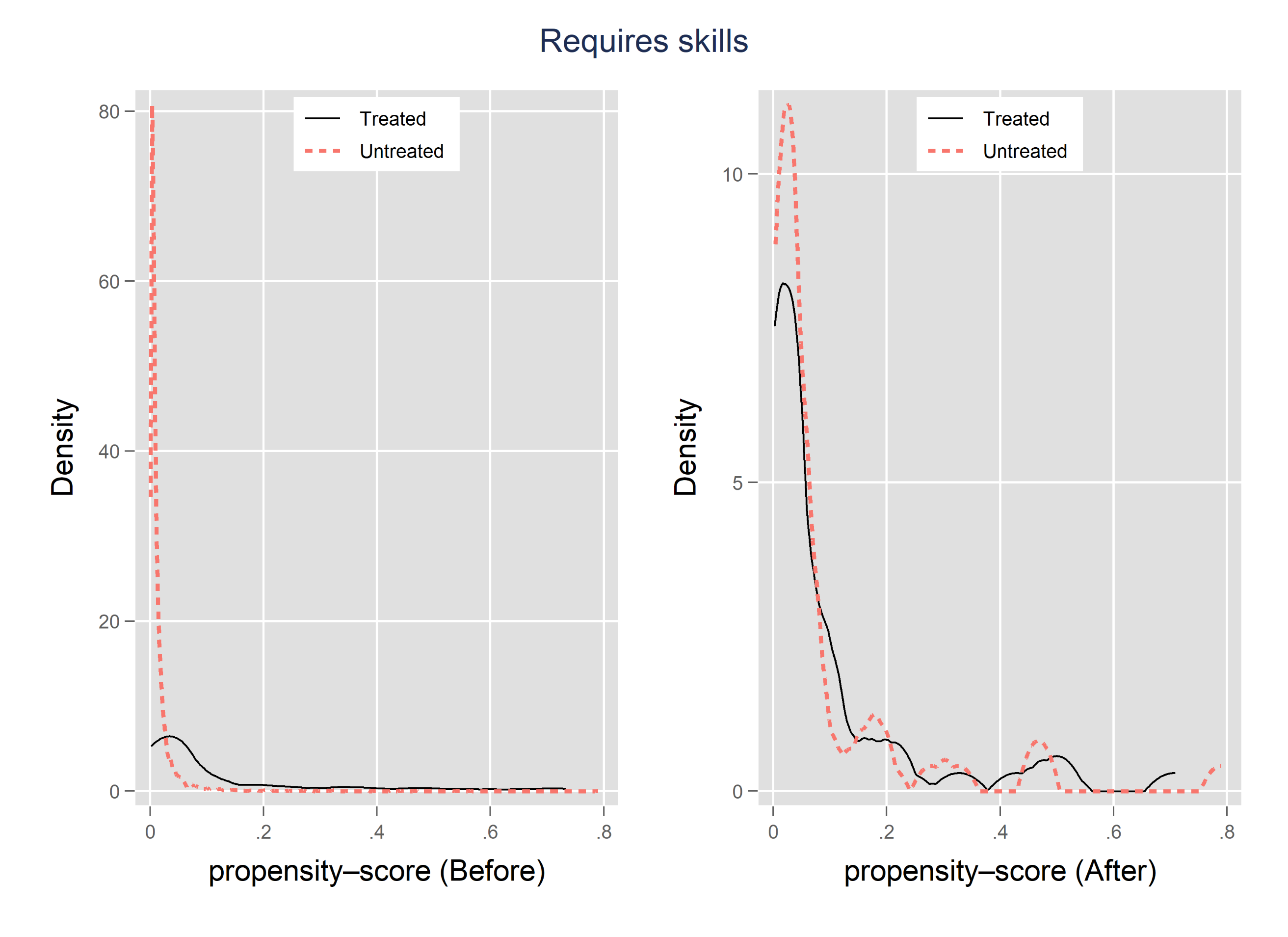

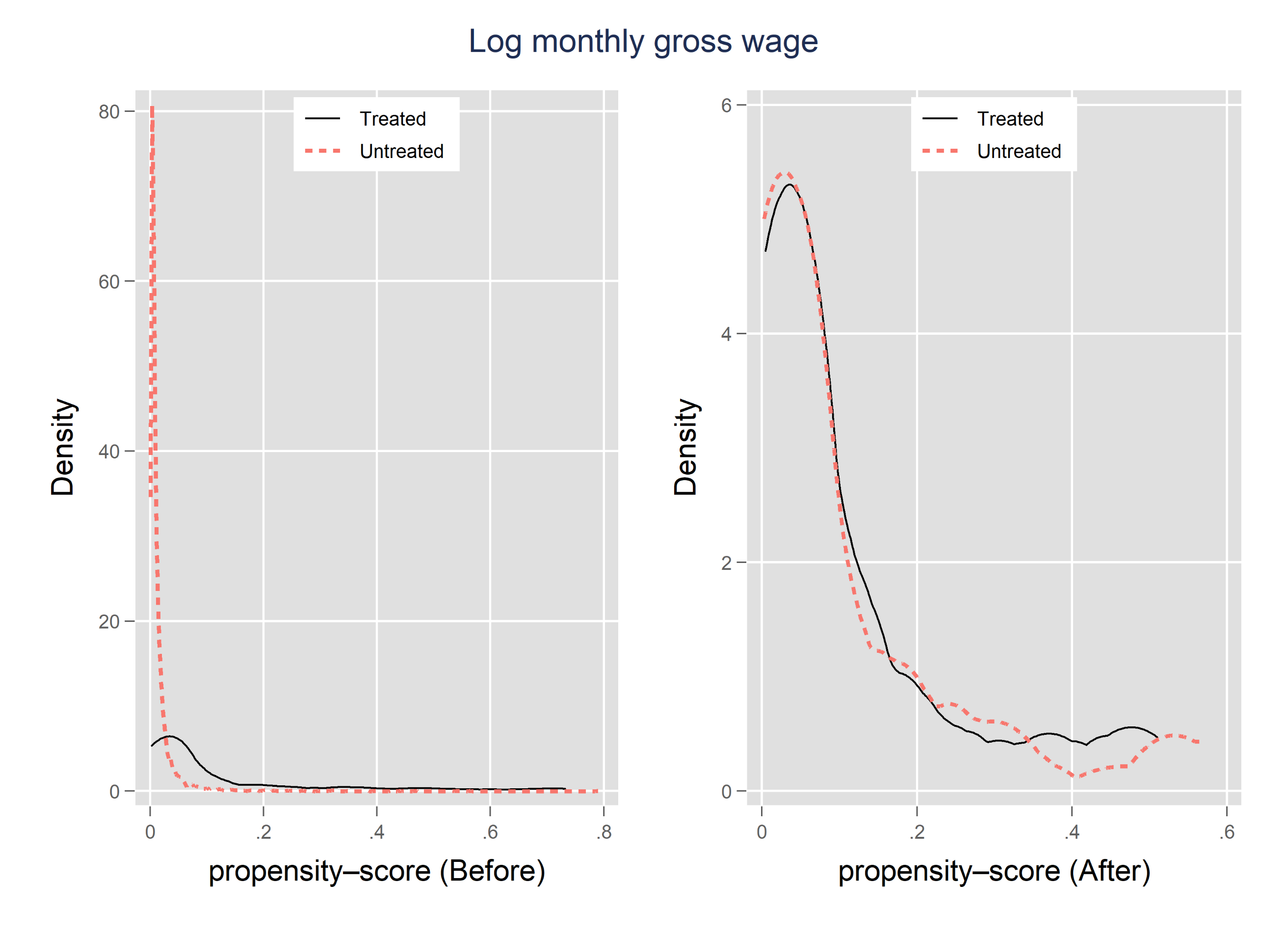


Table S.4.a: Results from Propensity Score estimation

|  | **Leuven and Sianesi (2003) NM 1:1** | | | | **Abadie and Imbens (2016) NM 1:1** | | | | **Abadie and Imbens (2016) NM 1:3** | | | |
| --- | --- | --- | --- | --- | --- | --- | --- | --- | --- | --- | --- | --- |
| ***All respondents*** | ATT | s.e. | T | N | ATT | s.e. | Z | N | ATT | s.e. | Z | N |
| NEET 19-20 | 0.081 | (0.075) | 1.08 | 4,943 | 0.081 | (0.066) | 1.23 | 4,943 | 0.129** | (0.053) | 2.45 | 4,943 |
| NEET 25-26 | 0.078 | (0.057) | 1.36 | 6,068 | 0.078 | (0.051) | 1.52 | 6,068 | 0.111** | (0.041) | 2.68 | 6,068 |
| Ever employed | -0.056 | (0.050) | -1.12 | 6,044 | -0.056 | (0.039) | -1.44 | 6,044 | -0.080** | (0.032) | -2.30 | 6,044 |
| Unemployment | 0.133 | (0.070) | 1.92 | 6,068 | 0.133** | (0.061) | 2.18 | 6,068 | 0.170*** | (0.050) | 3.33 | 6,068 |
| Econ. Hardship | 0.070 | (0.047) | 1.48 | 5,926 | 0.070 | (0.049) | 0.16 | 5,926 | 0.089** | (0.040) | 2.21 | 5,926 |
| ***Employed respondents*** |  |  |  |  |  |  |  |  |  |  |  |  |
| Routine occ. | 0.020 | (0.103) | 0.20 | 4,993 | 0.020 | (0.082) | 0.25 | 4,993 | 0.088 | (0.063) | 1.40 | 4,993 |
| Zero hrs contract | 0.082 | (0.068) | 1.20 | 4,964 | 0.082 | (0.058) | 1.40 | 4,964 | 0.075* | (0.044) | 1.71 | 4,964 |
| Full-time | -0.157* | (0.082) | -1.90 | 5,170 | -0.157** | (0.064) | -2.47 | 5,170 | -0.098 | (0.065) | -1.52 | 5,170 |
| Job requires skills | -0.020 | (0.103) | -0.20 | 5,023 | -0.020 | (0.098) | -0.21 | 5,023 | 0.00 | (0.071) | 0.00 | 5,023 |
| Log gross wage | -0.956** | (0.377) | -2.54 | 4,368 | -0.956*** | (0.294) | -3.25 | 4,368 | -0.696*** | (0.239) | -2.91 | 4,368 |

Note: Propensity Score matching estimation on common support area and with replacement; Actual nearest neighbour matches; Standard errors in parentheses; * p < 0.10, ** p < 0.05, *** p < 0.01; For the outcome NEET19 (wave 7) the sample size is a bit smaller than those for the outcomes at age 25 (wave 8) due to attrition. This is because some of the respondents who did not participate in wave 7 returned in wave 8.

Table S.4.b: Results from Inverse Probability of Treatment Weighting estimation

|  | **IPTW** | | | |
| --- | --- | --- | --- | --- |
| ***All respondents*** | ATT | s.e. | Z | N |
| NEET 19-20 | 0.140*** | (0.054) | 2.61 | 5,327 |
| NEET 25-26 | 0.152*** | (0.038) | 3.98 | 6,632 |
| Ever employed | -0.071** | (0.035) | -2.03 | 6,632 |
| Unemployment | 0.212*** | (0.043) | 4.95 | 6,632 |
| Econ. Hardship | 0.068* | (0.035) | 1.96 | 6,632 |
| ***Employed respondents*** |  |  |  |  |
| Routine occ. | 0.067 | (0.059) | 1.15 | 4,641 |
| Zero hrs contract | 0.060 | (0.047) | -0.32 | 4,641 |
| Full-time | -0.017 | (0.053) | 1.27 | 4,641 |
| Job requires skills | 0.070 | (0.062) | 1.14 | 4,641 |
| Log gross wage | -0.905*** | (0.213) | -4.25 | 4,641 |

Note: Standard errors in parentheses; * p < 0.10, ** p < 0.05, *** p < 0.01; For the outcome NEET19 (wave 7) the sample size is a bit smaller than those for the outcomes at age 25 (wave 8) due to attrition. This is because some of the respondents who did not participate in wave 7 returned in wave 8.

| Table S.4.c: Multivariate-distance nearest-neighbor matching (Mahalanobis distance) | | | | | | | | |
| --- | --- | --- | --- | --- | --- | --- | --- | --- |
|  | Coef. | s.e. | t | P>t | [95% Conf. | Interval] | Treated | Controls |
| ***All respondents*** |  |  |  |  |  |  |  |  |
| NEET19 | 0.18 | 0.06 | 3.11 | 0.00 | 0.07 | 0.30 | 62 | 450 |
| NEET25 | 0.13 | 0.04 | 2.99 | 0.00 | 0.05 | 0.22 | 83 | 648 |
| Ever employed | -0.05 | 0.04 | -1.35 | 0.18 | -0.13 | 0.02 | 85 | 643 |
| Unemployment | 0.20 | 0.05 | 3.71 | 0.00 | 0.09 | 0.30 | 78 | 648 |
| Economic hardship | 0.04 | 0.04 | 1.01 | 0.31 | -0.04 | 0.12 | 86 | 619 |
| ***Employed respondents*** |  |  |  |  |  |  |  |  |
| Routine occ. | 0.12 | 0.08 | 1.57 | 0.12 | -0.03 | 0.27 | 49 | 405 |
| Zero hrs contract | 0.11 | 0.05 | 2.12 | 0.03 | 0.01 | 0.22 | 49 | 404 |
| Full-time | -0.14 | 0.07 | -2.02 | 0.04 | -0.27 | 0.00 | 51 | 414 |
| Skills | 0.01 | 0.08 | 0.16 | 0.87 | -0.14 | 0.16 | 49 | 404 |
| Gross wage | -0.89 | 0.28 | -3.18 | 0.00 | -1.44 | -0.34 | 38 | 304 |

Note: Standard errors in parentheses; * p < 0.10, ** p < 0.05, *** p < 0.01; For the outcome NEET19 (wave 7) the sample size is a bit smaller than those for the outcomes at age 25 (wave 8) due to attrition. This is because some of the respondents who did not participate in wave 7 returned in wave 8.

Table S.5: Sensitivity analyses: Mantel-Haenszel and Rosenbaum tests

| **Outcomes** | **Result** | **Method** |
| --- | --- | --- |
| ***All respondents*** | |  |
| NEET 19 | [above 1.5 gamma] | Mantel-Haenszel |
| NEET 25 | [up to 1.45 gamma] | Mantel-Haenszel |
| Ever employed | [none - negative side mh: risk of underestimation] | Mantel-Haenszel |
| Unemployed | [above 1.5 gamma] | Mantel-Haenszel |
| How managing financially these days | [up to 1.05 gamma/none] | Mantel-Haenszel |
| ***Employed respondents*** | | Mantel-Haenszel |
| Routine occupations | [none] | Mantel-Haenszel |
| Zero-hour contract | [up to 1.35 gamma] | Mantel-Haenszel |
| Full-time job | [up to 1.10 gamma – negative side mh: risk of underestimation] | Mantel-Haenszel |
| Job requires skills | [none] | Mantel-Haenszel |
| (log) Gross wages | [above 1.5 gamma] | Rosenbaum |

Note: Gamma = how strong (in terms of Log-odds) should be the confounder to wash away our results; at p-mantel-haenszel at least below 10%; none = results are not robust to confounding; full tables are available upon request.

S.7: Absolute number of temporary suspensions and expulsions in England, 2006-2018


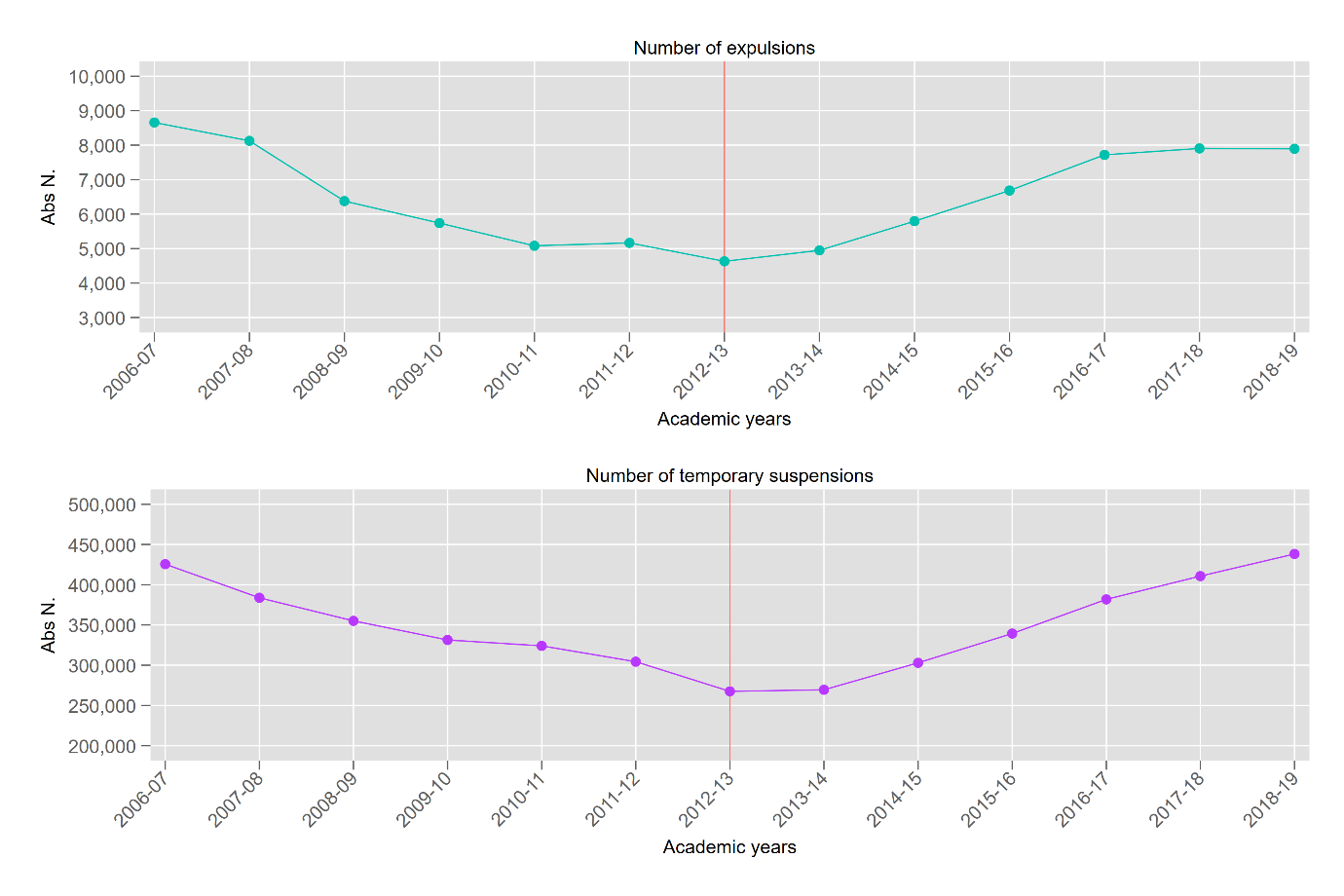


Source: UK Gov. School census, <https://explore-education-statistics.service.gov.uk/find-statistics/permanent-and-fixed-period-exclusions-in-england>; 2012/13 academic year coincides with educational reform regarding school exclusions.
